# Synergizing advanced materials and artificial intelligence for next-generation carbon capture, utilization, and storage (CCUS): a review

**DOI:** 10.1039/d5ra07338c

**Published:** 2026-01-12

**Authors:** Somia Mazhar, Muhammad Waseem Mumtaz, Mohamed El Oirdi, Hamid Mukhtar, Muhammad Asam Raza, Mohd Farhan, Mohammad Aatif, Ghazala Muteeb

**Affiliations:** a Department of Chemistry, University of Gujrat Gujrat Pakistan muhammad.waseem@uog.edu.pk; b Department of Biological Sciences, College of Science, King Faisal University Al-Ahsa 31982 Saudi Arabia meloirdi@kfu.edu.sa; c Institute of Industrial Biotechnology, GC University Lahore Lahore Pakistan; d Department of Chemistry, College of Science, King Faisal University Al-Ahsa 31982 Saudi Arabia mfarhan@kfu.edu.sa; e Department of Public Health, College of Applied Medical Sciences, King Faisal University Al-Ahsa 31982 Saudi Arabia maahmad@kfu.edu.sa; f Department of Nursing, College of Applied Medical Sciences, King Faisal University Al-Ahsa 31982 Saudi Arabia graza@kfu.edu.sa

## Abstract

The increasing rate of global carbon dioxide (CO_2_) emissions, mainly resulted from the industrial and energy sectors is a serious global challenge for climate stability. Carbon Capture, Utilization, and Storage (CCUS) technologies are being considered as important route to achieve the decarbonization objectives established in the Paris Agreement through reduction of CO_2_ levels in the atmosphere while allowing for its conversion to useful products. This review presents advancements in materials and technologies that are used to enhance the efficiency of CCUS process. Adsorbents based on biochar and nanomaterials, including carbon nanotubes, graphene derivatives, cellulose nanofibers, and nanoporous carbon, have significant CO_2_ capture potential, due to their tunable porosity and large surface area. In utilization metal–organic frameworks (MOFs), graphene-based catalysts, and single-atom catalysts (SACs) have promising selectivity in the electrochemical reduction of CO_2_ into fuels and chemicals in a closed carbon economy. For long-term storage, routes for secure and versatile sequestration include mineral carbonation, hydrate formation, and mixed-matrix membranes. Artificial Intelligence (AI) and Machine Learning (ML) enabled technology is increasingly crucial to the effectiveness of CCUS, not only in high-throughput material screening and predictive modeling for catalytic activity and plume migration forecasting, but also in system optimization. New digital tools, including digital twins, IoT-enabled monitoring, and life cycle assessments, increase the reliability, scalability, and sustainability of CCUS deployment. While there are many challenges remaining, especially with respect to cost, stability, and industrial scalability, CCUS can be seen as an emerging transformative technology towards net-zero energy transitions with advances occurring rapidly in synergy with materials science and digital intelligence.

## Abstract 2

This review provides insights into several material and AI/ML tools for CCUS technology.

## Graphical abstract

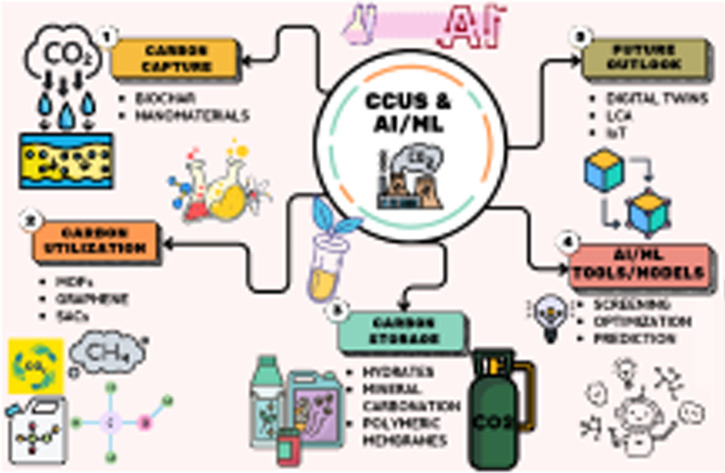

## 1 Introduction

**Fig. 1:**
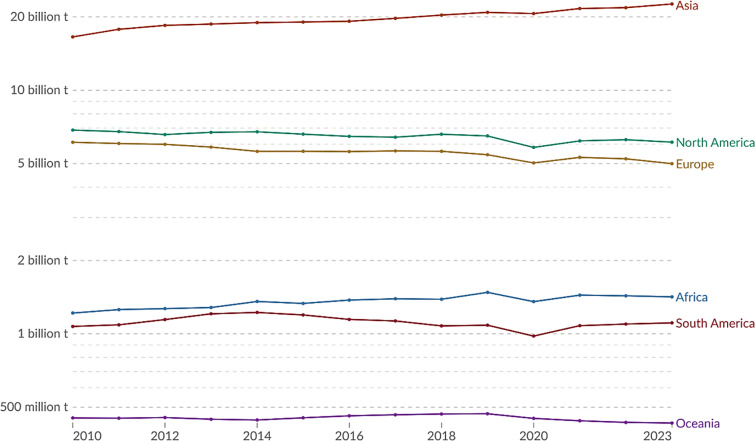
Annual CO_2_ emissions from fossil fuels and industry in all continents from 2010 to 2023. In this data, land-use practices such as deforestation or reforestation are not included.^5^ (Data source: Global Carbon Budget-2024.) “Data page: Annual CO_2_ emissions”, part of the following publication: Hannah Ritchie, Pablo Rosado, and Max Roser (2023) – “CO_2_ and Greenhouse Gas Emissions”. Data adapted from Global Carbon Project. Retrieved from https://archive.ourworldindata.org/20250716-155402/grapher/annual-CO2-emissions-per-country.html (archived on July 16, 2025) and licensed under Creative Commons (CC) by 4.0.

Human actions, such as burning fossil fuels, transportation, farming, and manufacturing fertilizers, steel, iron, and cement in bulk industrial production, are major sources of greenhouse gas (GHG) emissions.^1^ These GHG emissions contribute directly to rapidly accelerating the global warming, which is leading to drastic climate change. In 2018, global CO_2_ emissions were 67% of total GHG emissions.^2^ Looking ahead to the following years, the COVID-19 crisis had a positive impact on CO_2_ emissions. In comparison to CO_2_ emissions levels in 2019, a 7% decrease was recorded in 2020.^3^ However, in the post-COVID year, when social activities were back, a 6% increase in CO_2_ emissions was recorded as compared to the previous year.^4^ In 2022, 21.82 Gt of CO_2_ emissions were recorded from Asia due to territorial activities. 6.27 Gt from North America, 5.23 Gt from Europe, 1.43 Gt from Africa, 1.10 Gt from South America, and 0.43303 Gt from Oceania were also recorded in 2022, excluding the land-use changes.^5^ Moreover, the illustrative share of CO_2_ emissions of the continents from 2010 to 2023 is shown in Fig. 1.^5^

With these ongoing trends in CO_2_ emissions, the world will soon face tremendous CO_2_ accumulation in its atmosphere. Therefore, advanced technologies must be incorporated to bring down the elevated levels of CO_2_. One set of such technologies that attracted the attention of researchers in the past few years is Carbon Capture, Utilization, and Storage (CCUS) technology, because it helps to tackle a broad range of atmospheric problems that might include industrial CO_2_ emissions, CO_2_ accumulation in the atmosphere, an enigma of CO_2_ based products formation, and CO_2_ storage.^6^ This is because CCUS technology integrates various techniques that capture CO_2_ from emission sources, which can be industries or power plants. Afterwards, the fate of the captured CO_2_ lies at the nexus of either being utilized or stored.^7^

The CO_2_ can be captured either before or after the combustion of carbon-comprising fuels.^7^ However, when air reacts with fuels, NO_*x*_ can also be generated during these combustion processes. This can be avoided by using oxy-fuel combustion technology, in which pure oxygen reacts with fuel to carry out combustion, resulting in only CO_2_ along with water vapors.^8^ Post-combustion carbon capture from flue gases is best carried out by either the absorption/stripping method, which is based on Henry's law, or the adsorption/desorption method. While Knudsen diffusion principle and Fick's molecular diffusion form the basis of pre-combustion carbon capture through membranes.^9^ Zeolites, metal–organic frameworks (MOFs), silicon-based materials, covalent organic frameworks (COFs), and carbonaceous porous materials have been utilized for carbon storage. However, low CO_2_ partial pressure leads to poor selectivity, resulting in decreased adsorption performance. Many of these adsorbents become inactive at elevated temperatures due to their structural failures.^10^ The captured CO_2_ can be utilized in various ways. It can be employed for direct oil or gas recovery, foaming, fire extinguishers, dry ice, or welding. CO_2_-based solvents are also used for various organic reactions. In some organic reactions, CO_2_ is used as a reactant to produce many valuable chemical products. Desalination is another use of CO_2_.^11^ CO_2_ can be stored by: (i) geological storage method, (ii) oceanic storage method, or (iii) mineral storage method.^12^

**Fig. 2:**
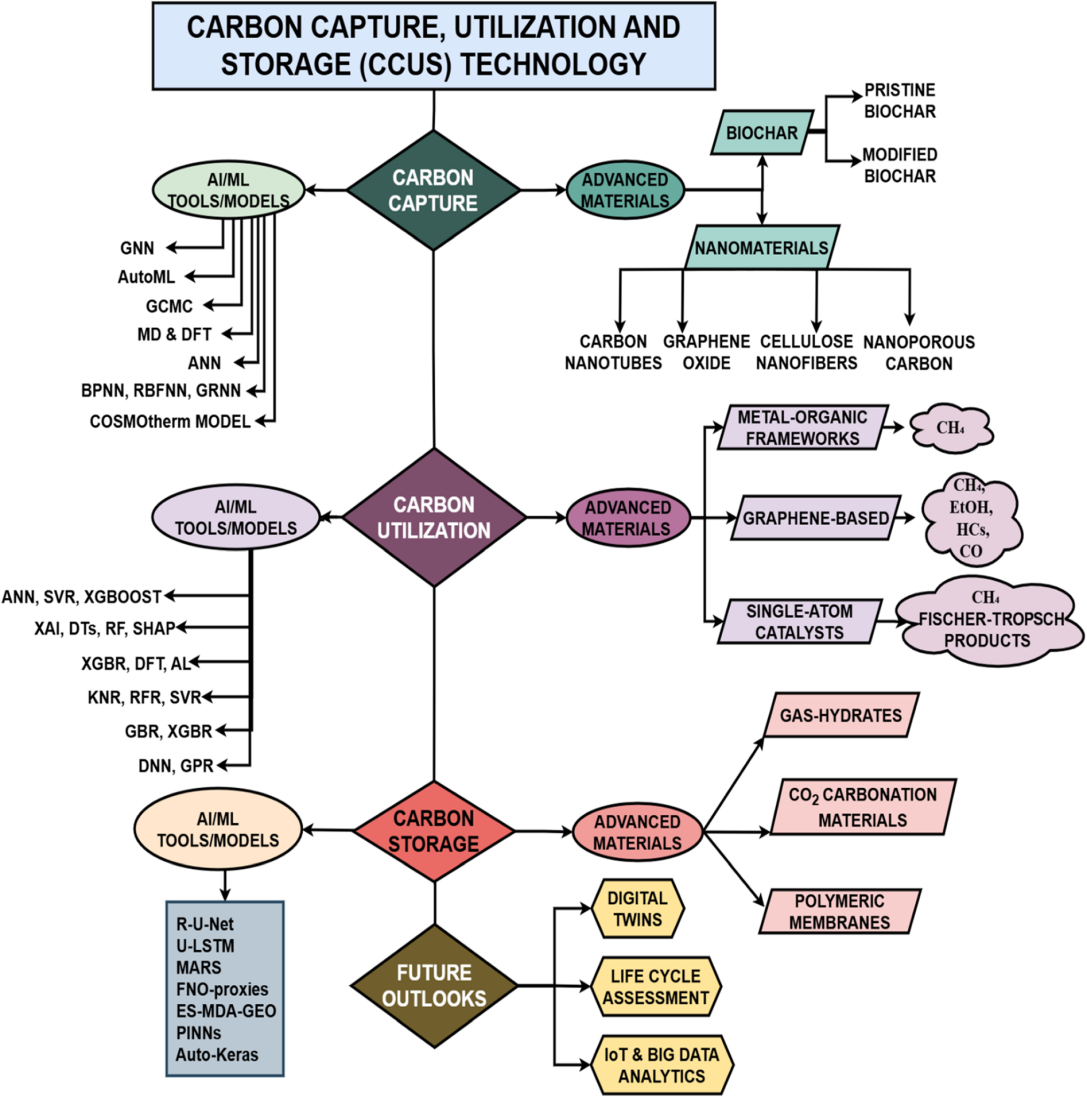
Advanced materials and AI/ML tools/models used in carbon capture, utilization and storage (CCUS) technology and some future outlooks.

A search of the Dimensions.ai database since 2020 yielded 14 527 articles under the entry “carbon capture, utilization, and storage (CCUS) technology.” In comparison, 7135 articles were retrieved using the term “advanced materials for CCUS technology,” while only 1851 articles appeared under “AI/ML for CCUS technology.” This imbalance highlights a significant research gap: although many papers report on CCUS in general, comparatively few examine the crucial contributions of advanced materials and AI/ML models. Consequently, this review discusses these two limited-but-potentially transformative areas of research to demonstrate their ability to improve the efficiency, scalability, and sustainability of CCUS processes. As illustrated in Fig. 2, the discussion begins with biochar-based adsorbents.^13^ It progresses to nanomaterials such as carbon nanotubes,^14^ graphene oxide,^15^ cellulose nanofibrils,^16^ and nanoporous carbon,^17^ each evaluated for their structural features, functional modifications, and CO_2_ adsorption capacities. The review then transitions to advanced catalytic systems, including metal–organic frameworks (MOFs),^18^ graphene-based materials,^19^ and single-atom catalysts (SACs),^20^ which enable the efficient electrochemical reduction of CO_2_ into fuels and value-added chemicals. Storage strategies such as hydrates,^21^ mineral carbonation,^22^ and polymeric membranes^23^ are also assessed from the perspective of scale-up and permanence. Along with materials, the study of artificial intelligence and machine learning tools has emerged as an area of emphasis in CCUS research, as it provides valuable assistance in accelerating the rate of material discovery, projecting catalytic performance, developing storage dynamics, and designing system-level integration.

Recent developments in 3D deep learning and point-cloud analysis have enhanced AI's ability to analyze complex spatial data structures, as shown in human–machine interaction and rehabilitation algorithms (*e.g.*, 3D graph deep learning for hand segmentation, and intelligent rehabilitation frameworks). These architectural developments exemplify transferable algorithm concepts that can help in the investigation and development of similar data-driven modeling in material design and CO_2_ process simulation.^24,25^

Collectively, the outline highlights how the convergence of advanced materials and AI/ML-driven frameworks marks a significant step toward the deployment of scalable, efficient, and sustainable CCUS.^26^ CCUS is undoubtedly an effective technique; however, the processes to capture CO_2_ from different sources, its conversion into valuable products, adequate transportation to storage areas, and optimizing storage sites require a high cost. Therefore, the development of various techniques that can carry out these protocols within a low budget with greater efficacy is demanded.^27^

## 2 Advanced materials for carbon capture

The development of advanced materials has emerged as a cornerstone of carbon capture research, offering novel adsorbents with enhanced surface properties, selectivity, and stability to trap CO_2_ from diverse emission sources efficiently. This section focuses on various biochar^13^ and nanomaterial-based^28^ entities for carbon capture. The CO_2_ adsorption capacity of different adsorbents is summarized in Table 1.

**Table 1:** Different materials for CO_2_ capture

S. no.	Adsorbent	Modification	CO_2_ adsorption capacity	Adsorption temperature	Adsorption pressure	Surface area	Ref.
1	Bamboo biochar (BBC-KOH)	Potassium hydroxide (KOH)	1.50 mmol g^−1^	25 °C	N/A	540.496 m^2^ g^−1^	29
2	Bamboo charcoal	Potassium hydroxide (KOH)	0.88 mmol g^−1^	25 °C	1 bar	526.36 m^2^ g^−1^	30
3	Bamboo biochar (BB)	Potassium hydroxide (KOH)	14.12 cm^3^ g^−1^ or 0.63 mmol g^−1^	0 °C	1 bar	7.83 m^2^ g^−1^	31
4	Orange peel biochar (OPB)	Potassium hydroxide (KOH)	22.83 cm^3^ g^−1^ or 1.01 mmol g^−1^	0 °C	1 bar	40.13 m^2^ g^−1^	31
5	Sawdust biochar	ZnCl_2_	1.58 mmol g^−1^	0 °C	1 bar	717.60 m^2^ g^−1^	32
6	MWCNT-COOH	N/A	0.1%	30 °C	0.9 bar	121 m^2^ g^−1^	14
7	MWCNT-SD-DETASi	DETASi	0.43 mmol g^−1^ (1.89%)	35 °C	1 bar	88 m^2^ g^−1^	14
8	MWCNT-OH-DETASi	DETASi	0.17 mmol g^−1^ (0.74%)	35 °C	1 bar	83 m^2^ g^−1^	14
9	MWCNT-COOH-DETASi	DETASi	0.33 mmol g^−1^ (2.11%)	30 °C	0.9 bar	74 m^2^ g^−1^	14
10	Graphene oxide	PAMAM/CNTs (0.6/0.12)	2.23 mmol g^−1^	25 °C	1 bar	126 m^2^ g^−1^	15
11	Graphene oxide	PAMAM/CNTs (0.2/0.03)	1.17 mmol g^−1^	25 °C	1 bar	115 m^2^ g^−1^	15
12	Graphene oxide	N/A	0.38 mmol g^−1^	25 °C	1 bar	74 m^2^ g^−1^	15
13	CNTs	N/A	0.032 mmol g^−1^	25 °C	N/A	N/A	33
14	CNTs	SiO_2_	0.670 mmol g^−1^	25 °C	N/A	N/A	33
15	CNF	3-Aminopropylmethyldiethoxysilane	2.26 mmol g^−1^	23 °C	N/A	7.5 m^2^ g^−1^	16
16	CNF	*N*-(2-Aminoethyl)-3-aminopropylmethyldiethoxysilane	1.91 mmol g^−1^	80 or 90 °C	N/A	51.8 m^2^ g^−1^	16
17	Nanoporous activated biocarbon	Potassium hydroxide (KOH)	30.4 mmol g^−1^	0 °C	30 bar	3106 m^2^ g^−1^	34
18	C-PP-700-1	S-doping with potassium persulfate	2.63 mmol g^−1^	25 °C	1 bar	810 m^2^ g^−1^	35
19	C-PP-750-1	S-doping with potassium persulfate	3.77 mmol g^−1^	0 °C	1 bar	866 m^2^ g^−1^	35
20	C-PP-750-1	S-doping with potassium persulfate	2.56 mmol g^−1^	25 °C	1 bar	866 m^2^ g^−1^	35
21	C-PP-750-2	S-doping with potassium persulfate	3.20 mmol g^−1^	25 °C	1 bar	849 m^2^ g^−1^	35
22	C-PP-750-3	S-doping with potassium persulfate	2.35 mmol g^−1^	25 °C	1 bar	866 m^2^ g^−1^	35
23	gNPCN-130	Inbuilt basic N-sites (48%), nanoconfinements by using the SBA15-130 template (9.15 nm pore size)	21.2 mmol g^−1^	0 °C	30 bar	466 m^2^ g^−1^	17
24	gNPCN-150	Inbuilt basic N-sites (48%), nanoconfinements by using the SBA15-150 template (11.24 nm pore size)	23.1 mmol g^−1^	0 °C	30 bar	553 m^2^ g^−1^	17
25	nTCN	N/A	4.6 mmol g^−1^	0 °C	30 bar	49 m^2^ g^−1^	17
26	Nanoporous carbon	Potassium hydroxide (KOH)	6.71 mmol g^−1^	0 °C	N/A	N/A	36
27	Nanoporous carbon	Potassium hydroxide (KOH)	4.214 mmol g^−1^	25 °C	N/A	N/A	36
28	N-doped nanoporous carbon	Potassium hydroxide (KOH)	6.40 mmol g^−1^	0 °C	1 bar	N/A	37
29	N-doped nanoporous carbon	Potassium hydroxide (KOH)	4.38 mmol g^−1^	25 °C	1 bar	N/A	37
30	N/O co-doped porous carbon	Na_2_CO_3_	1.95 mmol g^−1^	0 °C	1 bar	850.16 m^2^ g^−1^	38

### 2.1 Biochar adsorbents

Biochar is formed by pyrolysis at about <700 °C. It is one of the most advanced adsorbent used for carbon capture. Due to its high surface area, fine porosity, and remarkable volume, it is considered to be more effective and 10 times less costly than traditionally used carbon capture adsorbents. Furthermore, CO_2_ affinity can be enhanced by modifying the biochar surface with basic functional groups.^13^

#### 2.1.1 Synergistic activation and surface modification of biochar

Bamboo biochar (BBC-KOH) has been investigated to assess its CO_2_ capture capability, and it was found that it could adsorb 1.50 mmol g^−1^ of CO_2_ at 25 °C with low desorption temperature (80 °C). Bamboo biochar, when activated with KOH, was observed to exhibit enhanced surface area (from 374.42 m^2^ g^−1^ to 540.496 m^2^ g^−1^). It has been revealed that biochar before and after activation show that KOH also increased pore diameter from 28.812 angstroms to 38.496 angstroms. These findings spotlight the importance of synergistic enhancement through surface modification of biochar for CO_2_ capture; however, the adsorption capacity might decline with increasing temperature.^29^

The fact that the BET surface area of biochar adsorbents impacts their capture capacity can further be validated by a comparative study conducted with bamboo biochar (BB) and orange peel biochar (OPB). Both adsorbents were modified with KOH. BB adsorbed 14.12 cm^3^ g^−1^ or 0.63 mmol g^−1^, while OPB adsorbed 22.83 cm^3^ g^−1^ or 1.01 mmol g^−1^ of CO_2_. The difference arose due to the varying surface area that was recorded as 40.13 m^2^ g^−1^ for OPB and 7.83 m^2^ g^−1^ for BB.^31^

Apart from KOH, synergistic N-doping of a biochar adsorbent formed from corncob powder with K_2_CO_3_ and urea at approximately 800 °C was carried out, which showed an adsorption capacity of up to 5.69 mmol g^−1^ at 0 °C and 1 bar by positively affecting the activation energy between CO_2_ and the N-doped biochar. However, developing N-doped biochar material lacks mechanistic pathways that can control its pore size, and it is costly.^10^ K_2_CO_3_ yields are more enhanced, but KOH is preferable due to its simpler mechanistic approach.

#### 2.1.2 Limitations and counterproductive modifications

Modification of biochar adsorbents does not always directly relate to adsorption capacity. Instead, some modifications can lead to negative impacts. For example, bamboo charcoal, wood pallets, and coconut shells, when modified with H_3_PO_4_, introduce phosphorus-containing functional entities on their surface, making them slightly acidic, which in turn reduces their capture capacity. Similarly, modification of the aforementioned biochar adsorbents with ZnCl_2_ by agitating them with ZnCl_2_ for 24 hours decreased the carbon capture capacity of all three biochars due to the shrinkage of pore size during activation.^30^ However, bringing down the impregnation time to 12 hours for ZnCl_2_ modification might prevent structural collapse of biochar, as Kwon and Lee demonstrated that sawdust biochar, when modified with ZnCl_2_, adsorbed 1.58 mmol g^−1^ of CO_2_ at STP. Given the lower corrosivity and easier zinc recovery, therefore ZnCl_2_ is a suitable choice for modifying biochar adsorbents.^32^ The tetraethylenepentamine (TEPA) modification also decreases the adsorption capacity of biochar due to its large molecular size and structure.^39^ That's why modification should be done after choosing the most compatible reagent and only when needed, because even pristine biochar adsorbents can take up a significant proportion of CO_2_ if appropriately designed or have basic functional groups. In this context, a study shows that soybean straw biochar exhibits high basicity and can adsorb CO_2_ at a rate of 26.53–41.49 mg g^−1^, suggesting that alkalinity is a crucial factor for carbon capture.^40^

**Fig. 3:**
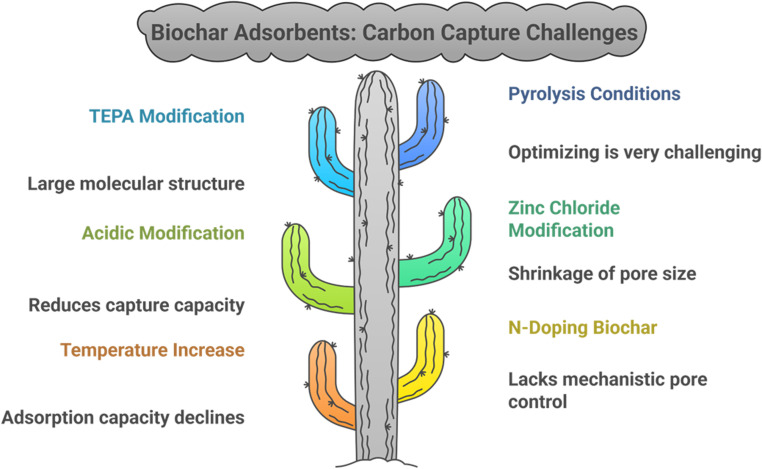
Challenges faced during biochar scaling for carbon capture.

While biochar adsorbents are promising for effective carbon capture, these are still in their development stage and require significant ongoing research because optimizing the conditions is one very challenging step due to the harsh conditions of pyrolysis and other hurdles, as illustrated in Fig. 3. Although scaling up biochar faces hurdles, a proper workup can make it possible in the upcoming years.

### 2.2 Nanomaterials for carbon capture

**Fig. 4:**
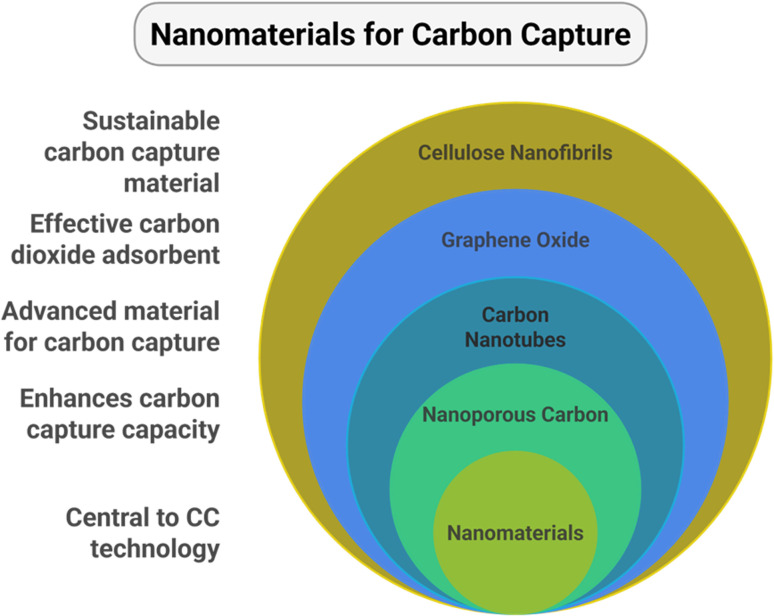
Nanomaterials for carbon capture and their benefits.

Scaling up advanced materials for CCUS technologies for large power plants requires low cost, greater availability, and increased functionality. The previously discussed materials are seen to lack the capabilities to overcome these challenges. However, synergistically engineered nanomaterials emerge as a class of compounds that could potentially accelerate progress in CCUS. Nanomaterials can be used in either pure form or as a hybrid. Functionalization of nanomaterials can be done with amines, carboxyls, or hydroxyls; however, amines have shown the highest CO_2_ capture capacity by making the adsorbents more selective towards CO_2_ and enhancing the hydrophobicity.^28^ The most prominent nanomaterials (as shown in Fig. 4) used for CO_2_ capture are carbon nanotubes,^14^ graphene oxide,^15^ cellulose nanofibrils,^16^ and nanoporous carbon.^17^

**Fig. 5:**
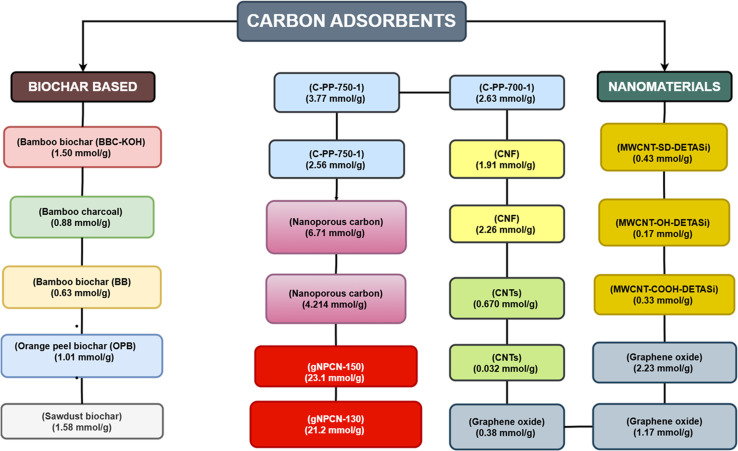
CO_2_ adsorption capacity of different adsorbents.^14–17,29–31,33,35,36^

#### 2.2.1 Carbon nanotubes (CNTs)

The primary advantage of using nanomaterials is CO_2_ retention in the adsorbents after adsorption, which can be seen in carbon nanotubes for CO_2_ capture. Hollow topological features provide large pore sizes to CNTs. Single-walled CNTs have the potential to adsorb up to 4 mmol g^−1^ of CO_2_, which is two times more than that of activated carbon.^41^ Multi-walled carbon nanotubes (MWCNTs), due to their remarkable strength, thermal conductivity, and electrical conductivity, as well as stable C–C linkages, can adsorb a significant volume of CO_2_.^42^

Functionalization does not always increase surface area or pore volume; rather, it provides binding functional groups in addition to chemisorption sites that synergistically enhance binding that can encompass more CO_2_ molecules. For example, amino-alkyl-silyl functionalization in three MWCNTs (MWCNT-SD, MWCNT-OH, and MWCNT-COOH) with *N*^1^-(3-trimethoxysilylpropyl)diethylenetriamine (DETASi) showed that the specific surface area of MWCNTs was changed from 112, 103, and 121 m^2^ g^−1^ to 88, 83, and 74 m^2^ g^−1^, respectively. The findings indicate that when the specific surface area was reduced to 74 m^2^ g^−1^, the adsorption capacity (0.33 mmol g^−1^) increased to its maximum compared to all other adsorbents. At the same time, pristine MWCNT-SD and MWCNT-OH were unable to adsorb CO_2_, despite having a large surface area of 112 and 103 m^2^ g^−1^, respectively. This occurred due to amine groups that provided chemisorption sites for the adsorbate, and the homogeneity of organosilicon groups increased the Henry's constant for distributed adsorption.^14^ However, the modifiers that simultaneously provide basic functional groups and increase surface area are more effective. Various combinations of modifiers can be used together to achieve this objective.

#### 2.2.2 Graphene oxide (GO) and modifiers

To carry out the modification resulting in basic functional groups and increase surface area, Pruna and co-workers modified graphene oxide (GO) aerogels with polyamidoamine (PAMAM) dendrimers and CNTs in different ratios that significantly enhanced the cross-linking abilities of GO. The synergistic effect of modifiers increased the surface area of the adsorbents from 74 m^2^ g^−1^ to 115 m^2^ g^−1^ and 126 m^2^ g^−1^, and adsorbed 1.17 and 2.23 mmol g^−1^ of CO_2_ at 25 °C when PAMAM/CNTs were 0.2/0.03 and 0.6/0.12, respectively. This work suggested that the scalability and durability of CO_2_ adsorbents can be increased by using these modifications in different concentrations because PAMAM improves CO_2_ adsorption by providing amine (basic) groups, while CNTs provide accessibility by reducing GO sheets stacking and acting as spacers to optimize gas adsorption.^15^

CNTs, either used independently or in combination with other adsorbents as modifiers, have their cohesive nature always affecting the fluidization by agglomeration, which in turn adversely affects CO_2_ capture. This can be avoided by using SiO_2_ nanoparticles for modification. For example, pure CNTs were modified to monoethanolamine–silicon–CNT (MEA–Si–CNT) by using SiO_2_ nanoparticles that enhanced the CO_2_ capture capacity from being negligible (0.032 mmol g^−1^) to 0.670 mmol g^−1^. This work potentially provides a practical solution for industrial applications, as the fluidization problem has not been addressed before this research work, but it requires surface area investigation as well.^33^ Nanoparticles of TiO_2_ are another class of modifiers to change wettability and decrease interfacial tension (IFT) of the adsorbents.^43^ Moreover, the selectivity of graphene can also be enhanced by using crown nanopores.^44^

#### 2.2.3 Cellulose nanofibers (CNFs)

Aforementioned adsorbents are of great importance; however, the challenge of toxicity still prevails. The cellulose nanofibers (CNF) are bio-degradable and non-toxic nano-adsorbents. These are usually functionalized with silane derivatives, which significantly reduce the BET surface area of CNF adsorbents but increase the selectivity of CO_2_, thereby enhancing the overall adsorption process. *N*-(2-Aminoethyl)-3-aminopropylmethyldiethoxysilane modified CNF could adsorb 1.91 mmol g^−1^ of CO_2_, even with 51.8 m^2^ g^−1^ BET surface area.^45^ Silane groups can undergo self-polymerization on the CNF surface. Still, the addition of acetic acid can prevent this by catalyzing the reaction of silane with silanol on the cellulose surface.^16^ One significant challenge in carbon capture technology is achieving the required balance between microporosity and mesoporosity in the adsorbent, which decreases the adsorption capacity. The previously stated nanomaterials do not fulfil this gap.

#### 2.2.4 Nanoporous carbon

Nanoporous carbon is another class of adsorbents that effectively fills the gap of porosity by providing an exceptionally large surface area and tremendous pressure withstanding ability even at elevated temperatures. All these properties render them the best adsorbents for CO_2_. For example, nanoporous activated biocarbon from alligator weed with a surface area up to 3106 m^2^ g^−1^ adsorbed 30.4 mmol g^−1^ of CO_2_.^34^ Nanoporous carbon prepared by pyrolysis (700 and 750 °C) of coconut shell and sulfur-doped with potassium persulfate, can adsorb CO_2_ at 0 °C and 25 °C, as shown in Table 1.^35^ And for pressure bearing ability, even at 30 bar, nanoporous carbon nitride modified with nanoconfinements of porous silica templates could adsorb 21.2 and 23.1 mmol g^−1^ of CO_2_.^17^ The efficient adsorption capacity of nanoporous carbon is attributed to the presence of oxygen-based functional groups, which are more selective towards CO_2_, and its tailored porosity. *In situ* N-doping of nanoporous carbon can also provide an oxygen-containing framework with enhanced microporosity that also assists in electrostatic adsorption.^37^

### 2.3 Polymeric membranes

Mixed matrix membranes (MMMs) have also demonstrated significant potential for enhancing CO_2_ capture owing to their superior permeability and selectivity compared to pure polymers. Their ability to surpass the Robeson upper bound has made them particularly attractive for post-combustion capture and natural gas sweetening applications. Functionalization strategies such as hydrogel-coating, PEGylation, and carboxylation further improve CNT dispersion within the polymer matrix, mitigate interfacial defects, and establish preferential CO_2_ transport pathways.^23^ For instance, Pebax MH 1657 MMMs incorporating NIPAM-coated multi-walled carbon nanotubes (NIPAM-MWCNTs) achieved a 35% increase in CO_2_ permeability and an 11% improvement in selectivity relative to the neat polymer, proving these effective for natural gas and flue gas separation. While these membranes improved capture efficiency, their storage capacity is not reported, leaving uncertainties regarding large-scale deployment potential.^46^ Building upon this, MMMs fabricated from Polymer of Intrinsic Microporosity (PIM-1) with PEG-functionalized MWCNTs achieved even higher performance gains, showing a 53.5% enhancement in CO_2_ permeability and an 18.8% increase in CO_2_/N_2_ selectivity. These improvements were most prominent at low filler loadings (0.5–2 wt%), highlighting the critical role of optimized dispersion. However, beyond 2 wt% CNT content, agglomeration occurred, which hindered gas transport and partially offset the advantages, underscoring the challenge of maintaining uniform distribution at higher loadings.^47^ Hence, different membranes can be utilized for CO_2_ storage, but the challenges of cost and scalability persist.

These materials are highly effective for CO_2_ capture; however, future directions can be explored by developing composites that incorporate basic functional groups, which are more selective towards CO_2_, and enhance both micro- and mesoporosity of the adsorbent. Thermal stability is another factor that needs to be addressed if scaling up to industrial power plants has to be carried out. Table 1 and Fig. 5 summarize different adsorbents for carbon capture.

### 2.4 Different carbon capture processes and advanced materials

**Fig. 6:**
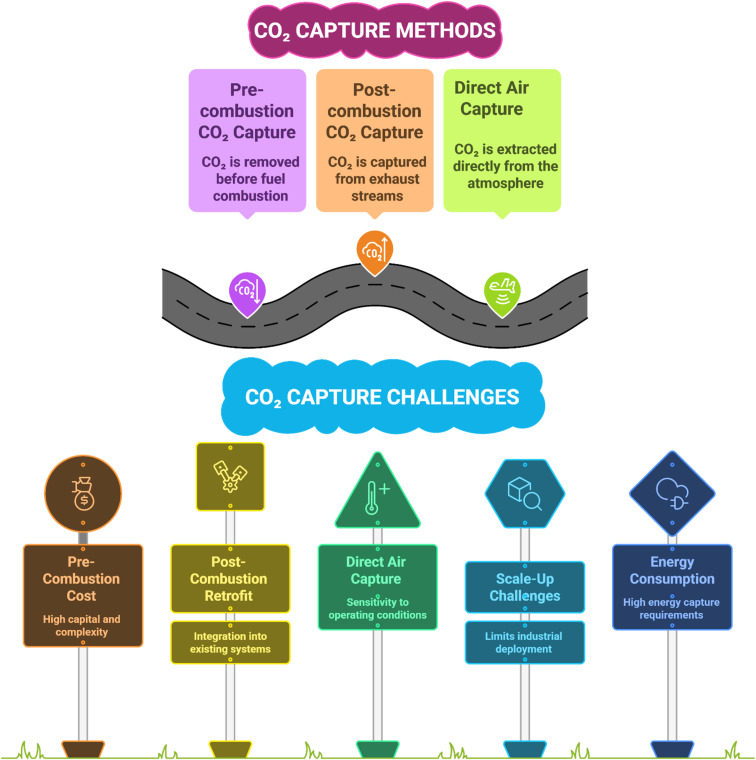
Carbon capture methods and some challenges.

Carbon capture can be carried out by three different methods: (1) pre-combustion carbon capture, (2) post-combustion carbon capture, and (3) Direct Air Capture (DAC), as shown in Fig. 6.

#### 2.4.1 Pre-combustion carbon capture

In pre-combustion CO_2_ capture, carbon dioxide is captured before the fossil feedstock is combusted. Pre-combustion involves gasifying a fossil feedstock to a syngas (CO + H_2_), converting CO to CO_2_ by the water–gas shift reaction, and separating CO_2_ from an H_2_-rich stream. Pre-combustion CO_2_ capture is primarily carried out at large-scale Integrated Gasification Combined Cycle (IGCC) power plants and hydrogen production facilities, providing a path to low-carbon and hydrogen gas, but with limited implementation due to high capital cost and complexity of the process.^48^

Pre-combustion CO_2_ capture has been analyzed in Integrated Gasification Combined Cycle systems, where fossil fuels are converted into syngas (CO + H_2_O) and CO_2_ is separated from hydrogen. Researchers examined novel physical solvents, such as PEG siloxane 1 and [aPy][Tf_2_N], and ionic liquids, like [P_2228_][CNPyr], to capture CO_2_ streams of 100 kmol h^−1^. They reported a CO_2_ capture cost of $93.86 per tonne CO_2_ using an ionic liquid system and found that chemical-specific solvents and ionic liquids can improve capture rates and offer economically viable alternatives to traditional methods at pre-combustion conditions of high temperature and high pressure.^49^ A separate study examined Selexol, a solvent used to remove CO_2_ and H_2_S at the same time, from shifted syngas in pre-combustion carbon capture for IGCC systems. Using Honeywell UniSim R400 with a Henry's law solubility model and the Peng–Robinson EOS for vapor-phase behavior, the researchers found that at high CO_2_ pressures (12–20 bar) and with a shared solvent circulation system, a two-stage Selexol process could achieve ≥99% hydrogen recovery, 90–95% CO_2_ capture, and CO_2_ purity below 20 ppm.^50^

#### 2.4.2 Post-combustion carbon capture

Post-combustion CO_2_ capture is a well-suited approach to add on to the exhaust of a variety of existing industrial and energy systems, *i.e.*, emissions from cement plants, steel production, power, or mobile point sources.^51^

Different materials have been used for CO_2_ uptake after combustion. He *et al.* (2019) demonstrated that ionic liquid-grafted activated carbon is better than ionic liquid-impregnated activated carbon and raw activated carbon in terms of post-combustion CO_2_ uptake and CO_2_/N_2_ selectivity, because of less pore blockage and better moisture retention of the carbon's porosity. The grafted system also provides stability and recyclability under consecutive cycles. The performance, however, was still a function of ionic liquid loading. The performance metrics indicated were also not quantified or benchmarked according to standard flue-gas conditions.^52^ A recent assessment of post-combustion CO_2_ capture indicated that amine-based chemical absorption is still the only fully commercial approach. This is based on large-scale operations, such as the Benfield process, which captures ∼700 000 tonne CO_2_ per year. Physical solvents, such as Rectisol, also exhibit high-purity CO_2_ recovery, with large-scale facilities capturing 7–8.4 million tonnes per year, with proper pressure–temperature conditions in place. The review notes that some MOF adsorbents, such as Mg/DOBDC (∼5.95 mmol g^−1^), have good potential for low-pressure uptake of post-combustion streams, but high material costs and challenges regarding scale-up during industrial conditions remain an obstacle for deployment.^53^ Coconut and palm shells were modified into activated carbon. This modification them into porous compounds with surface areas of 132.76 and 101.92 m^2^ g^−1^, respectively, and pore sizes of about 8 nm. These properties make suitable materials for post-combustion CO_2_ capture. Activation resulted in a rise of fixed carbon and decreased volatiles, and thus, cleaner carbon matrices with increased microporosity were found.^54^ And some recent studies have also focused on the porous carbon materials (PCMs) for post-combustion CO_2_ capture, by focusing on biomass-derived precursors, chemical activation, and heteroatom doping, *etc.*^55^

All these materials exhibit notable activity; however, an alternative study on ionic liquid has demonstrated that 1-ethyl-3-methylimidazolium cation and the bis(trifluoromethylsulfonyl)imide anion [emim][Tf_2_N] in an oxy-fuel combustion plant can yield extremely low-energy CO_2_ capture, providing 112.27 kWh per tonne CO_2_, 98.2% desorption efficiency, and 99.9% CO_2_ purity at 0.01 MPa. The authors also demonstrated that the solvent's physical properties (ionic liquid density, heat capacity, and thermal conductivity) strongly control circulation rate and heat-exchanger requirements by varying these properties. Knowledge of the physical properties of these materials is therefore useful when designing a high-performance CO_2_-capture solvent in any capture configuration.^56^

#### 2.4.3 Direct air capture (DAC)

Regarding direct air capture, one study describes Rail-based DAC, which incorporates CO_2_ capturing units on trains, and incorporates regenerative braking and onboard solar to power flexible sorbents (amines, MOFs, electro-swing quinones) without external energy. Each train can remove 75–105 tonne CO_2_ per day with projected total costs of ≈$45 per tonne, and could ultimately offer a global capacity of up to 7.8 Gt per year by 2075. This approach minimizes emissions, infrastructure, and land area associated with DAC while providing a scalable and energy-efficient option for DAC.^57^ In another study, a distributed DAC system is demonstrated and integrates carbon nanofiber (CNF)-based filters into building heating, ventilation, and air conditioning (HVAC) systems. The CNF adsorbent reaches a theoretical production of 4 mmol CO_2_ per g, which could allow a theoretical removal potential of 596 Mt CO_2_ per year globally. Regeneration occurs through either solar, thermal, or electrothermal Joule heating, which greatly limits energy demand. The life cycle assessment showed carbon removal efficiency of 92.1% and techno-economic analysis showed costs of carbon removal is $209–668 per tonne CO_2_, highlighting a scalable, low-carbon, and compatible DAC solution for existing infrastructure.^58^ Another study looked at enzyme-assisted DAC that utilized carbonic anhydrase (CA) to increase absorption of CO_2_ into aqueous carbonate solutions. Under DAC-relevant conditions (0.04% CO_2_), adding micromolar concentrations of CA increased the absorption rate by a factor of three and increased capture efficiency from ∼20% to 60% (even at higher gas flow rates). Compared to KOH solutions, adding 1 µM carbonic anhydrase slows down the fast hydroxide reaction and shows that enzyme-based carbonate sorbents could make direct air capture (DAC) possible with lower regeneration energy (about 7–9 GJ per ton of CO_2_ avoided). This approach could capture CO_2_ more efficiently than standard hydroxide-based processes.^59^ Overall, several advances can be seen in pre-combustion, post-combustion, and direct air capture technologies, *i.e.*, in materials development, process design, and improved energy efficiencies, as demonstrated through the use of custom solvents, ionic liquids, and new adsorbents that enable higher CO_2_ capture rates and overall purity, as presented in Table 2 below.

**Table 2:** Summary of recent advances in pre-combustion, post-combustion, and direct air carbon capture technologies, including materials, processes, and key performance metrics

Carbon capture type	Technology/material	Process/setup	Key performance metrics/findings	Ref.
Pre-combustion	Selexol (physical solvent)	Dual-stage CO_2_/H_2_S removal from shifted syngas in IGCC; simulated in Honeywell UniSim R400 with Henry's law + Peng–Robinson EOS	≥99% H_2_ recovery, 90–95% CO_2_ capture, CO_2_ purity < 20 ppm, energy consumption aligns with DOE NETL	50
	Ionic liquids ([P_2228_][CNPyr])	CO_2_ capture from syngas streams (∼100 kmol h^−1^)	Capture cost $93.86 per tonne CO_2_; high capture efficiency at high T and P	49
	PEG siloxane 1, [aPy][Tf_2_N]	Novel physical solvents evaluated	Enhanced CO_2_ separation performance	49
Post-combustion	IL-grafted activated carbon	Flue-gas CO_2_/N_2_ separation	Improved CO_2_ uptake and selectivity; reduced pore blockage; good recyclability	52
	Amine-based chemical absorption (Benfield process)	Commercial post-combustion capture	∼700 000 tonne CO_2_ per year captured	53
	Physical solvents (Rectisol)	Industrial CO_2_ separation	7–8.4 million tonne CO_2_ per year under suitable conditions	53
	MOFs (*e.g.*, Mg/DOBDC)	Low-pressure CO_2_ uptake	Uptake ∼5.95 mmol g^−1^	53
	Biomass-derived activated carbons	Post-combustion CO_2_ adsorption	Surface areas: 132.76 and 101.92 m^2^ g^−1^; pore size ∼ 8 nm; enhanced microporosity	54
	Porous carbon materials (PCMs), polymers, foams	Adsorbents for CO_2_	Enhanced porosity and adsorption performance; heteroatom doping improves performance	55
	Ionic liquid [emim][Tf_2_N]	Oxy-fuel combustion	CO_2_ capture energy 112.27 kWh per tonne, 98.2% desorption efficiency, 99.9% CO_2_ purity; IL properties (density, heat capacity, thermal conductivity) influence performance	56
Direct air capture (DAC)	Rail-based DAC (amines, MOFs, electro-swing quinones)	DAC units on trains, powered by regenerative braking and solar	75–105 tonne CO_2_ per day per train; cost ≈$45 per tonne; global potential 7.8 Gt per year by 2075; reduces land use and grid burden	57
	CNF-based filters	Distributed DAC integrated in HVAC	CO_2_ capacity 4 mmol g^−1^; global removal potential 596 Mt per year; 92.1% life-cycle carbon removal efficiency; cost $209–668 per tonne CO_2_	58
	Enzyme-assisted DAC (carbonic anhydrase, CA)	CO_2_ absorption in aqueous carbonate solutions	Under 0.04% CO_2_: absorption rate tripled; capture efficiency 20 → 60%; 1 µM CA compensates KOH kinetics; lower regeneration energy (∼7–9 GJ per tonne CO_2_ avoided)	59

## 3 Advanced materials for carbon utilization

**Fig. 7:**
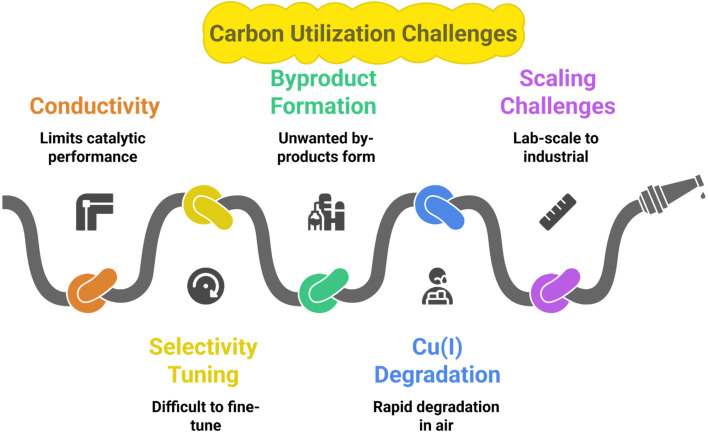
Challenges faced during carbon utilization.

CO_2_, after being captured, can be utilized in various ways. This carbon utilization can be chemical (into chemicals, materials, fuels), biological (into fuels, feed, fertilizers, or food), or geological (into petroleum, natural gas, water, or minerals).^60^ The following are various materials that utilize carbon in different ways. Table 3 summarizes various catalysts/systems along with their carbon utilization efficiency, products, and limitations, and Fig. 7 Illustrates some challenges being faced during carbon utilization, as explained in the following sections.

**Table 3:** CO_2_ utilization and limitations of systems

S. no.	Catalyst/system	Key feature	Main product(s)	FE/current density	Limitation(s)	Ref.
1	Cu-THQ, Cu-HHTP	Square-planar CuO_4_	CH_4_	<2% FE	Low CO activation, poor selectivity	18
2	Cu-DBC (CuO_5_)	π-backbonding → *CO → *CHO	CH_4_	56% FE	Still moderate efficiency	18
3	HATNA-Cu-MOF	π–π stacking stability	CH_4_	78% FE	CH_4_ exclusivity low	61
4	Cu_4_-MFU-4l	Trigonal Cu(i)–N sites	CH_4_	81% FE	Cu(i) is unstable in air	62
5	NNU-33(H)	–OH ligands, cuprophilic interactions	CH_4_	82% FE, 391 mA cm^−2^	Requires high stability	63
6	NNU-32	—	CH_4_	55% FE, 384 mA cm^−2^	Lower selectivity	63
7	Cuobpy nanosheets	Morphology control	CH_4_	82% FE	Scalability issue	64
8	Adenine-Cu-MOF	Shape-dependent	CH_4_	50% FE	Mid selectivity	65
9	2D-vc-MOF(Cu)	Vacancies	CH_4_	65% → 32% FE	Fragile structure	66
10	Cu_2_O@CuHHTP	MOF-derived	CH_4_	73% FE, 10.8 mA cm^−2^	Below best MOFs	67
11	CuNPs from MOF-74	Single Cu NPs	CH_4_	>50% FE	Moderate selectivity	68
12	Cu–N-C (from Cu-BTC)	Temp. tuning of Cu–N_*x*_	CH_4_/C_2_H_4_	13.9–38.6% FE	Trade-off selectivity	69
13	N-doped graphene + ionic liquid	Imidazolium cations	CH_4_	93.5% FE, high current	Conventional instability solved	70
14	Cu NPs on N-graphene	N sites steer *CO coupling	C_2+_ alcohols (EtOH, *n*-PrOH)	—	C_2+_ enhanced	71 and 72
15	Au NPs (∼2.4 nm) on RGO	Defect sites + amine additives	CO	59–75% FE	Product selectivity controlled	73
16	Ag–graphene–N-C fibers	Hybrid electrode	Acetate, EtOH, CO, H_2_	—	Moderate selectivity	19
17	RGO–CdS (Ag-NW enhanced)	Light-driven activity	CO, CH_4_	—	Charge recombination improved	74
18	Ir–Co SAC	Stabilized HCP Co phase	Fischer–Tropsch products	Reduced CH_4_ sel. 10% → 2.7%	High stability (1200 h)	20
19	Cu-SAC in C_3_N_4_	Single Cu sites	CH_4_	68% FE	Moderate	75
20	Cu–N_2_O_2_ SAC	N,O-coordinated	CH_4_	78% FE, 40 mA cm^−2^	—	76

### 3.1 Metal–organic frameworks (MOFs) for methane production

For CO_2_ electrochemical reduction (CO_2_ER) to methane, MOFs play a vital role. For example, starting from low-selectivity Cu-THQ and Cu-HHTP with square-planar CuO_4_ sites that delivered <2% faradaic efficiency (FE) at −1.4 V. However, their limitation in activating and hydrogenating CO intermediates directs towards the synthesis of geometrically tuned Cu-DBC (CuO_5_), which strengthens π-back-bonding and promotes *CO → *CHO, reaching 56% FE at −1.4 V. Still, these systems have moderate efficiency.^18^ To boost selectivity and efficiency, HATNA-Cu-MOF leverages π–π stacking for structural stability and achieves 78% FE at −1.5 V, yet requires further improvement in CH_4_ selectivity.^61^

Cu_4_-MFU-4l (Cu-BTDD derivative) introduces trigonal Cu(i)–N_3_ sites to control hydrogenation to CH_4_, delivering 81% FE at −1.2 V in neutral media; however, Cu(i) air sensitivity constrains its practicality towards stability.^62^ Addressing stability and current, NNU-33(H) employs –OH ligands and strong cuprophilic interactions to stabilize intermediates and suppress HER, reaching 82% FE at −0.9 V with 391 mA cm^−2^, whereas NNU-32 has comparatively lower selectivity (55% FE) and current (384 mA cm^−2^).^63^

#### 3.1.1 Morphological and derivative enhancements

Morphology control further helps: Cuobpy single-layer nanosheets expose more sites to push FE to 82% at −1.4 V, but handling ultrathin layers at a large scale is challenging.^64^ While adenine-based Cu-MOFs show shape-dependent gains up to 50% FE at −1.6 V (nanosheets best), they remain mid-range in selectivity, leaving room for improvement.^65^ Potential sensitivity appears in 2D-vc-MOF(Cu), where FE falls from 65% at −1.2 V to 32% at −1.4 V due to structural changes, highlighting operational fragility.^66^

To reconcile stability with selectivity, MOF-derived catalysts with controlled single-type sites emerge. Cu_2_O@CuHHTP achieves 73% FE and current 10.8 mA cm^−2^ at −1.4 V, improving robustness yet falling short of the best FEs.^67^ Isolated CuNPs from MOF-74 suppress C_2_ coupling and surpass commercial NPs with >50% FE at −1.3 V, but still moderate selectivity.^68^

By combining current and selectivity, Cu/CeO_2_@C from MOF precursors achieves 80.3% FE with and current 138.6 mA cm^−2^, utilizing a carbon encapsulation strategy that prevents over-reduction and addresses both durability along with rate simultaneously.^77^ In summary, composition/spacing and tuning in Cu–N-C that is derived from Cu(BTC)(H_2_O)_3_ results in a change in FE from 13.9% to 38.6% (800–900 °C). The temperature modulation of Cu–N_*x*_ decides the formation and nature of products (C_2_H_4_*vs.* CH_4_), but the selectivity is still lower than Cu(i)-MOFs, underscoring the trade-off between air-stable derivation and peak FE.^69^

#### 3.1.2 Summary and synergistic findings

These findings suggest that MOFs offer a unique combination of adjustable pore sizes, high surface areas, and custom catalytic centers, which lower the energy barrier for CO_2_ activation and preferentially direct hydrogenation toward methane. The synergistic interplay between structural design, active metal centers, and coordination environment allows MOFs to achieve high CH_4_ yield and FEs even at the low temperatures typically available from renewable electrical sources, thus operating effectively within a closed carbon cycle. Additionally, fine-tuning of the coordination environment minimizes hydrogen and carbon monoxide side reactions, a strategy that several Cu-based MOFs in the literature have successfully demonstrated. However, the plan is hindered by several key limitations: the frameworks typically exhibit low intrinsic conductivity, limiting the catalytic performance of the unmodified MOFs; selectivity remains difficult to fine-tune; unwanted by-products continue to form; Cu(i) centers with the best performance suffer from rapid degradation upon exposure to air; and the established promising lab-scale behaviors have yet to be continuously and reliably scaled and embedded within industrial systems.

### 3.2 Graphene-based materials for CO_2_ER

Besides methane, graphene-based materials can produce valuable products like CO, acetate, ethanol, hydrogen (H_2_), and other hydrocarbons. For example, an ionic-liquid-treated N-doped graphene/carbon paper helps stabilize CO_2_ using imidazolium cations, reduces the voltage needed, suppresses H_2_ formation, and achieves methane with 93.5% faradaic efficiency. It also provides stable, high current output, solving the selectivity and stability problems seen in conventional systems.^70^ Using the improved activity gained from doping, copper nanoparticles placed on N-doped graphene use the nitrogen sites to guide *CO toward forming *OC–COH. This helps produce C_2+_ alcohols like ethanol and *n*-propanol. Hence, these materials overcome the usual problem of low C_2+_ formation by improving C–C coupling on carbon-based supports.^71,72^

#### 3.2.1 Synergistic hybrid systems

To further tune CO selectivity and kinetics, Au (∼2.4 nm) on reduced graphene oxide used defect sites and amine additives to raise CO selectivity from 32–60% up to 59–75%, showing that molecular interfacial modifiers on graphene supports can systematically boost CO formation, addressing the need for product-specific selectivity control.^73^ Extending toward multi-carbon products, an Ag–graphene–N-doped carbon fiber electrode yielded acetate, ethanol, CO, and H_2_ across −0.4 to −1.0 V *vs.* RHE, with the graphene-containing architecture enhancing ethanol faradaic efficiency over controls, bridging the gap between CO-selective systems and C-2 product generation *via* synergistic support coupling.^19^ In addition, RGO–CdS systems (confirmed by Ag nanowires) took advantage of graphene's conductivity and π–π interactions with CO_2_ to improve yields of CO/CH_4_ and suppress charge recombination, closing the remaining gap of inefficient light-driven activation of CO_2_ with enhanced charge transport and CO_2_ adsorption.^74^

Conventional CO_2_ reduction catalysts work slowly and lack selectivity and stability. Graphene-based materials improve performance because they are conductive, tunable, and robust. Although pure graphene is inactive, doping and structural design make it reactive and selective. Its large surface area and strong support for nanoparticles improve charge flow, prevent clumping, increase active sites, and enhance CO_2_ adsorption and overall CO_2_ reduction efficiency.

### 3.3 Single-atom catalysts (SACs)

Catalysis as a field is undergoing a paradigm shift, where the design of catalysts has become central.^78^ The core principle lies in constructing active sites and tailoring their surrounding environments to control catalytic activity, selectivity, and stability.^79^ With advances in nanoscience, the scale of the active phase has evolved from nanoscale and sub-nanoscale dimensions to the single-atom dimension, leading to the emergence of single-atom catalysts (SACs).^80^

The concept of SACs was first introduced in 2011 by researchers who developed platinum SACs *via* a co-precipitation method for carbon monoxide oxidation.^81^ SACs are particularly attractive because of their maximum atom utilization and unique structural properties, and with the progress of controllable synthesis strategies, they have found applications in toxic gas handling, fuel cells, and CO_2_ utilization.^82,83^

Due to their unique physico-chemical properties and the flexibility of adjustable coordination environments, SACs often exhibit superior performance compared to conventional nanocatalysts in CO_2_ utilization.^82^ For example, Co-based catalysts used in Fischer–Tropsch synthesis (FTS) suffer from methane overproduction and water-induced oxidation. By stabilizing the hexagonal close-packed (HCP) phase of Co nanoparticles, an Ir–Co SAC successfully reduced methane selectivity from 10% to 2.7% and extended durability to over 1200 hours, far exceeding the 200 hours achieved by conventional Co catalysts.^20^ Many non-toxic C-1 chemicals can be produced by CO_2_ using SACs.^84^ Likewise, in the CO_2_ electrochemical reduction reaction (CRR), the high overpotential to activate the C=O bond combined with multiple competing pathways typically leads to poor conversion and selectivity. SACs outperform other catalysis because of their high atomic utilization efficiency and uniformity in the active site structure, allowing for reliable interaction with substrates, increasing selectivity.^82^ For example, Cu-supported catalyst within carbon nitride matrices converted CO_2_ to CH_4_ with 68% faradaic efficiency at −0.84 V during an electrochemical reduction process.^75^ Similarly, N, O-coordinated SACs, such as Cu–N_2_O_2_, achieved 78% faradaic efficiency while converting CO_2_ to CH_4_ at a current density of approximately 40 mA cm^−2^.^76^

Despite the advantages, the rational design of SACs remains highly challenging because of the vast combinatorial space of possible metal–support combinations and coordination environments. Traditional trial-and-error approaches are insufficient for effectively exploring these possibilities.

#### 3.3.1 Theoretical correlation between single-atom active sites and product selectivity

The catalytic selectivity of single-atom catalysts (SACs) in CO_2_ electroreduction is fundamentally governed by the electronic structure and coordination environment of the isolated metal center. According to transition-state theory, the rate constant for an elementary reaction step is *k* = *A*e^−*E*_a_/*RT*^, where the activation energy barrier *E*_a_ determines both the rate and relative probability of competing pathways. Distinct single-atom sites (*e.g.*, Cu–N_4_, Fe–N_4_, Ni–N_4_) exhibit different *E*_a_ values for key intermediates such as *COOH, *CHO, and *OCH_3_, which define the branching between CO, CH_4_, and CH_3_OH products.^85^

Density functional theory (DFT) calculations thus provide a quantitative mapping between reaction energy barriers and product selectivity, where lower-barrier pathways dominate under applied potentials. By comparing adsorption energies, charge distribution, and projected density of states, the optimal coordination environment can be theoretically deduced as the one that stabilizes desired intermediates while suppressing undesired hydrogen evolution.^86^

Moreover, rational adjustment of coordination geometry reveals that the optimal active-site configuration is the one that stabilizes target intermediates while inhibiting side reactions such as the hydrogen evolution reaction (HER).^87^

**Fig. 8:**
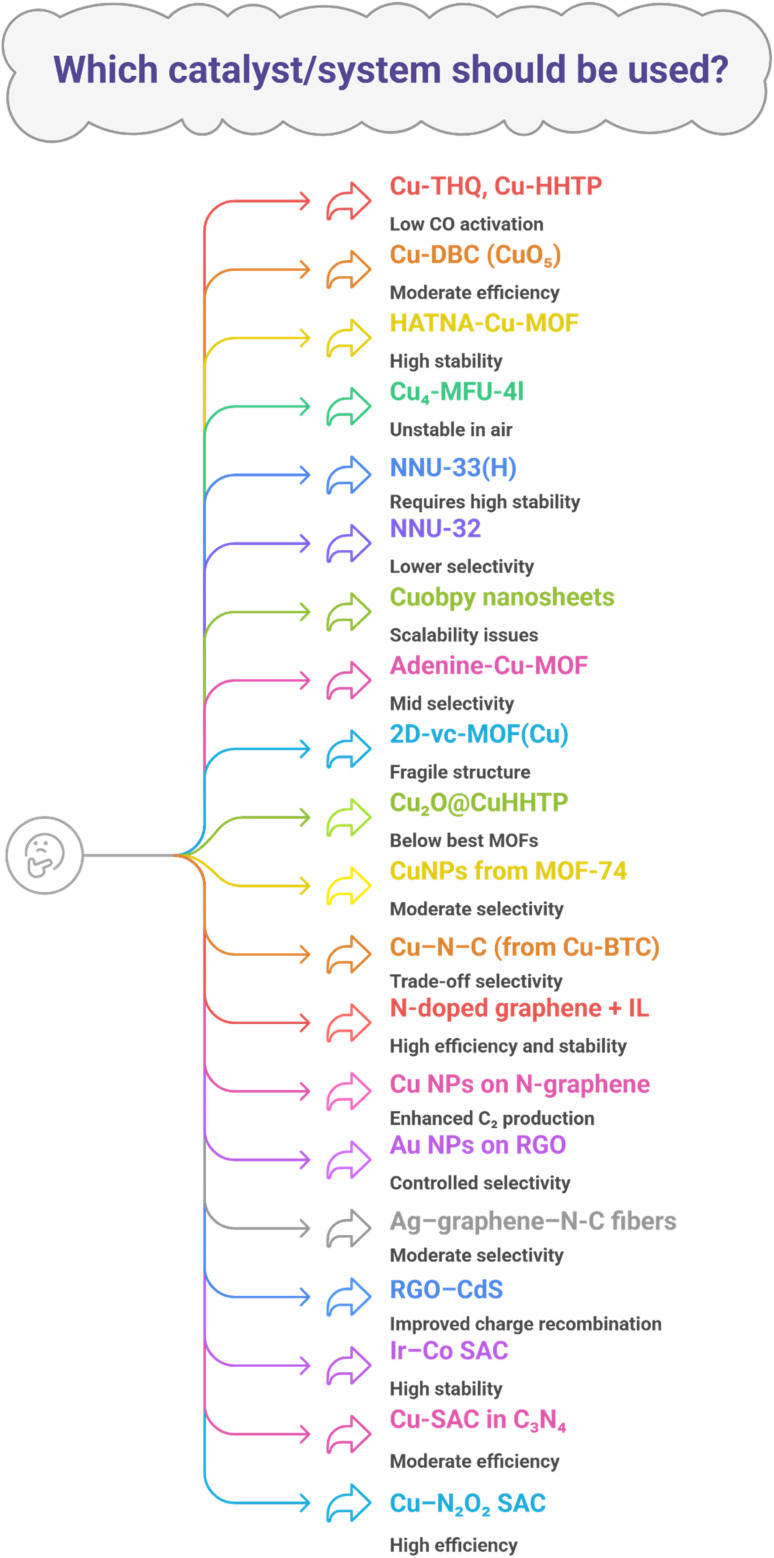
Key features of various materials that can aid in selecting the most suitable system for carbon utilization.

This section demonstrates how different systems behave in terms of carbon utilization. However, choosing the most exquisite system is quite a laborious task. Fig. 8 demonstrates key features of various materials that can help when choosing the best system for carbon utilization. Table 3 further validates the results by summarizing key aspects.

## 4 Advanced materials for carbon storage

**Fig. 9:**
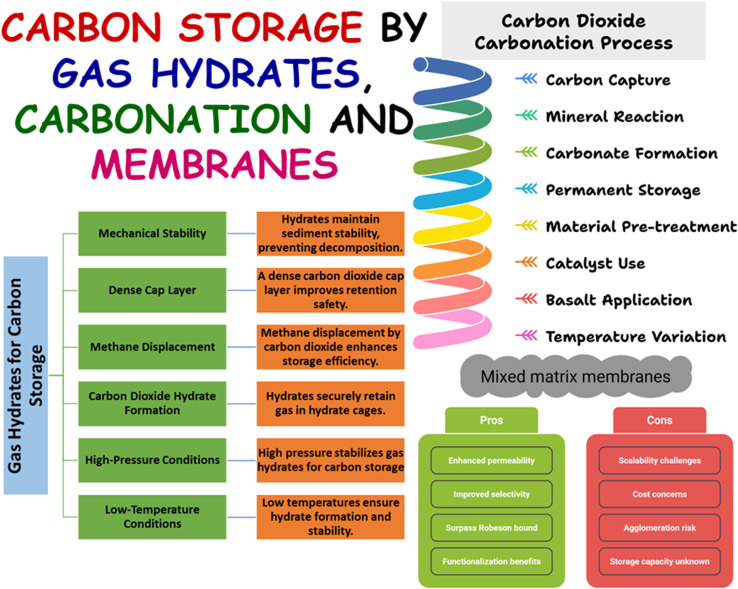
Key points of carbon storage by gas hydrates, mineral carbonation, and membranes.

A diverse array of advanced materials has been explored for CO_2_ storage, ranging from naturally occurring hydrates^21^ and mineral carbonation systems^22^ to engineered polymeric membranes,^23^ each offering unique mechanisms, advantages, and challenges for scalable carbon management, as shown in Fig. 9.

### 4.1 Hydrates for carbon storage

Gas hydrates are crystalline water-ice cage structures encapsulating gas molecules (*e.g.*, methane or ethane). They are generally produced under high-pressure and low-temperature conditions, such as in permafrost areas or marine deep-water areas below 500 m depth, where natural gas hydrates (NGHs) are abundant, energy-rich, and thus suitable as CO_2_ storage candidates.^21^ CO_2_ can also exist as a liquid or hydrate in offshore conditions, where the denser liquid form aids in maintaining safe storage.^88^ A major advantage is that CO_2_ can replace methane inside hydrate cages because it is more stable under those conditions. As a result, CO_2_ stays trapped safely after it pushes out the methane.^89^ Furthermore, some of the injected CO_2_ may form a dense cap layer, enhancing the system's overall retention safety. Simultaneously, the hydrates that develop contribute to the bond between sediment grains, preserving the mechanical stability that might otherwise be compromised by hydrate decomposition. The synergy of both capping and clathration enables a concurrent withdrawal of methane and the containment of carbon dioxide, thereby achieving energy yield and a controlled carbon sink within the same intact pore-constrained framework.^90^

### 4.2 CO_2_ carbonation materials

The carbon storage method that captures CO_2_ by making it react with minerals to give off stable and solid carbonates for permanent storage is regarded as mineral carbonation or CO_2_ carbonation.^22^ CO_2_ carbonation is tailored through various materials and conditions; for example, serpentine, which is a hydrated silicate material, is thermally pre-treated to convert it into olivine and becomes compatible for aqueous mineral carbonation.^91^ Sodium bicarbonate can act as a catalyst/buffer and, as a result, boost olivine carbonation by almost 60% within 5 hours by maintaining pH within a specific range (mild to slightly basic, 7.2–9.0), while NaCl could only carry out olivine carbonation up to 20%.^92^ Wang *et al.* (2019) reported the formation of magnesium and iron carbonate mixtures with simultaneous Ni/Co recovery by using olivine carbonation.^93^ While the formation of nesquenhonite, hydromagnesite, and dypinigite was also reported in a previous study, by using Mg(OH)_2_ as carbonation material, however transportation of Mg(OH)_2_ still causes problems.^94^ In another study, Segamat basalt, enriched in olivine and pyroxene with Ca- and Mg-bearing phases, demonstrates strong potential for CO_2_ mineral carbonation, with natural calcite formation confirming reactivity. A storage capacity of approximately 91 520 tons CO_2_ has been estimated, supported by favorable porosity, mineral composition, and proximity to emission sources, though large mineral requirements and reliance on assumed storage ratios highlight material intensity and scale-up limitations.^95^ Mostly, the previous studies require low temperatures for mineral carbonation. Mazaheri *et al.* (2025) carried out mineral carbonation at about 26–80 °C at different stages, by utilizing Segamat basalt and converted CO_2_ into dolomite [CaMg(CO_3_)_2_], siderite (FeCO_3_), calcite/vaterite (CaCO_3_), magnesite, and ankerite.^96^ Thus, CO_2_ carbonation emerges as a versatile CO_2_ storage technique, capable of generating stable carbonates under different conditions; however, scalability and material intensity remain critical challenges for its future application.

## 5 Material link synergistic adaptation matrix for CCUS systems

To create a connected carbon capture, utilization, and storage chain, we must not only develop new materials, but our materials must work together to facilitate the transport of CO_2_, the capture, conversion, and storage process. The materials including amines-based adsorbents, MOFs, graphene composites, single-atom catalysts, mineral carbonates, and mixed-matrix membranes show how phenomena like interfacial compatibility, reaction coupling, and stability enhancements can collectively work together to establish an efficient closed-loop carbon cycle. A unified matrix of these materials is presented in Table 4 to visualize their collaborative linkages and synergistic enhancements across the CCUS process chain.

**Table 4:** Material link synergistic adaptation matrix for CCUS systems

Material/system	Application stage(s)	Synergistic mechanism	Key performance/outcome	Ref.
Bamboo biochar (BBC-KOH)	Capture	Synergistic modification: KOH activation + surface basic groups	1.50 mmol g^−1^ CO_2_ at 25 °C; ↑SA (374.42 → 540.496 m^2^ g^−1^), ↑pore diameter	29
Corncob N-doped biochar (K_2_CO_3_ + urea)	Capture	Synergistic N-doping + activation	Up to 5.69 mmol g^−1^ at 0 °C, 1 bar	10
Sawdust ZnCl_2_-modified biochar	Capture	Synergistic timing control (12 h *vs.* 24 h)	1.58 mmol g^−1^ at STP (12 h); 24 h can cause pore collapse	30 and 32
Soybean straw biochar	Capture	Synergistic surface alkalinity	26.53–41.49 mg g^−1^ uptake	40
CNTs (functionalized)	Capture and utilization (support)	Synergistic functionalization (amines, SiO_2_, MEA)	SWCNT up to 4 mmol g^−1^; functionalized MWCNTs show improved chemisorption despite ↓SA; MEA–Si–CNT 0.670 mmol g^−1^	14, 41 and 33
GO + PAMAM + CNTs	Capture and utilization (support)	Synergistic coupling: amine groups + spacers	Adsorbed 1.17 and 2.23 mmol g^−1^; improved accessibility and catalytic support	15
CNFs (silane-modified)	Capture	Synergistic bio-functionalization	1.91 mmol g^−1^ (51.8 m^2^ g^−1^)	16 and 45
Nanoporous carbon (N-/O-doped)	Capture and high-pressure storage	Synergistic micropore tailoring + doping	Up to 30.4 mmol g^−1^ (3106 m^2^ g^−1^); 21–23 mmol g^−1^ at high pressure in some systems	17, 34 and 37
MOFs (Cu variants)	Capture/utilization	Synergistic coordination and ligand tuning	CH_4_ FE 56–82% depending on design (Cu-DBC, HATNA-Cu, NNU-33(H), *etc.*)	18, 61–63, 67,68,70 and 77
MOF-derived Cu/CeO_2_@C	Utilization	Synergistic encapsulation	FE ∼ 80.3%; *j*CH_4_ ≈ 138.6 mA cm^−2^	77
Graphene-based (N-doped, rGO + Au)	Utilization	Synergistic electronic tuning and interfacial modifiers	CH_4_ FE 93.5% (ionic-liquid N-doped graphene); FECO 59–75% (Au/rGO)	70–73
SACs (Ir–Co, Cu–N_2_O_2_, Cu in C_3_N_4_)	Utilization	Synergistic atom–support coupling	Ir–Co reduces undesirable CH_4_ (improved durability > 1200 h); Cu–N_2_O_2_ FE 78%; Cu@C_3_N_4_ FE 68%	20,75,76 and 82–84

## 6 Role of AI and ML in CCUS technology

**Fig. 10:**
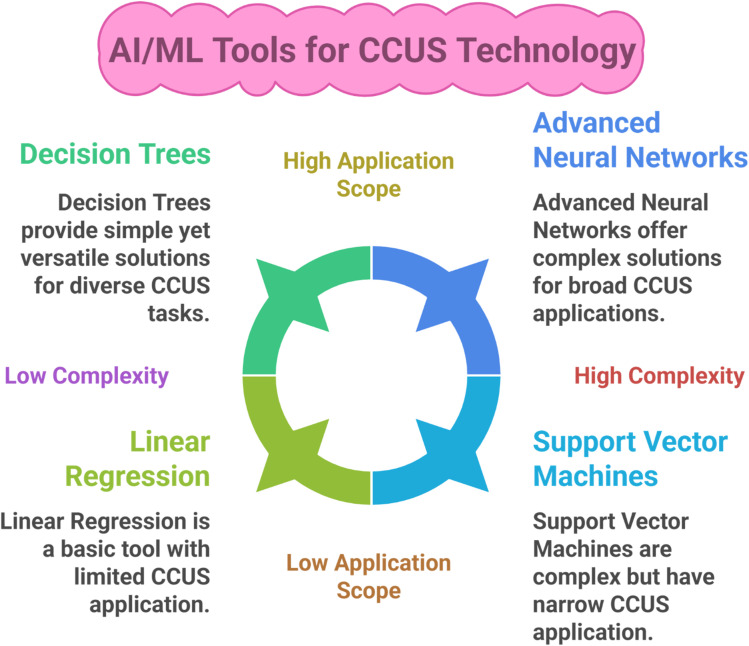
An overview of AI/ML in CCUS technology.

CCUS materials are the epitome of the decarbonization steps undertaken to meet the Paris Agreement targets. Therefore, their synthesis, optimization, and utilization require more advanced technologies that could enhance overall performance beyond measures. One such strategy is the integration of Artificial Intelligence and Machine Learning with CCUS technology. ML is a subset of AI, and it comprises various statistical tools and advanced algorithms that can be utilized to effectively optimize, predict, classify, or cluster the data for material synthesis that is used in CCUS technology (Fig. 10).^26^ This section covers various AI/ML tools/models for carbon capture, utilization, and storage, while Table 5 summarizes the AI/ML tools/models for CCUS technology, and Fig. 10 Gives an illustrative overview of the process.

**Table 5:** AI/ML tools/models for CCUS

S. no.	AI/ML tool/model	Purpose	Outcomes	Ref.
1	Graph neural networks (GNN), AutoML	Predict CO_2_ adsorption capacity/selectivity; optimize structure–performance mappings of MOFs/ILs	Shortened R&D cycles and reduced costs by identifying pore chemistries and topologies	97
2	ML-accelerated workflow (GCMC + ML prediction + mixture simulation)	Screen large chemical spaces of COFs	Screened 268 687 COFs; identified 3D COFs (∼1.0 nm pores) and 2D COFs (imide linkages, fluoro groups) as best CO_2_ adsorbents	98
3	ML models trained on DFT	Predict thermophysical properties (heat capacity) of porous materials	Achieved 95% accuracy for zeolites; errors of 3.2 J kg^−1^ K^−1^ for MFs, 2.5 J kg^−1^ K^−1^ for COFs	99
4	Self-supervised deep learning; ML with property-labeled descriptors	Generate material embeddings; predict thermal properties	Uncovered structure–function relationships; accelerated screening for CO_2_ capture materials	100
5	ANN-based ML	Predict CO_2_ capture performance and CO_2_/N_2_ selectivity in MOFs	Stable models; adsorption capacity predictions more accurate than selectivity; surface area and pore size positively correlated with CO_2_ uptake	101
6	ML models trained to refine DFT energy calculations	Correct limitations of MD and DFT (cost, precision)	Reduced cost; improved accuracy and scalability for larger systems	102
7	BPNN and RBFNN	Predict CO_2_ solubility in 12 amine solvents	Both achieved good accuracy; BPNN outperformed RBFNN and 8 other literature models	103
8	BPNN and GRNN	Predict CO_2_ solubility, density, and viscosity of solvents	BPNN best for solubility; GRNN more precise for density and viscosity (*e.g.*, potassium lysinate)	104
9	Ensemble random forest and decision tree	Predict CO_2_ solubility in 185 ILs	RF achieved MAE = 0.04; DT achieved MAE = 0.10; more accurate than COSMOtherm model	105
10	LSSVM, DT, RF, MLR (with GA-MLR feature selection)	Predict CO_2_ solubility in ILs (using thermodynamic and structural descriptors)	RF and DT showed strong agreement with experiments; pressure was the most influential factor; HOMO–LUMO fraction was the key descriptor	106
11	Proxy ML model	Optimize CCS/EOR cases	Predicted 2916 cases with ∼92% accuracy	101
12	ANN, SVR, XGBoost	Surrogate modeling for CO_2_-EOR	Optimized injection strategies and economic prediction; more accurate than traditional AI simulations	107
13	Random forest	Predict cumulative CO_2_ injected in EOR	Identified operational parameters as key drivers	107
14	Deep learning + multi-physics simulations	Optimize SOEC performance under thermal-neutral conditions	Validated SOEC performance under varying thermal parameters	108
15	Extreme gradient boosting regression (XGBR)	Predict/screen electrocatalysts for CO_2_ reduction	Successfully identified suitable electrocatalysts	109
16	Active ML + DFT	Identify Cu–Al catalysts	Discovered catalysts reducing CO_2_ to ethylene	110
17	ML regression trained on DFT	Predict active catalysts for CO_2_RR	Identified Cu–Al alloy with >80% faradaic efficiency *vs.* 66% for pure Cu	110
18	ML regression (trained on DFT C–H dissociation barriers)	Screen single-atom alloy catalysts	Identified Ir/Ni catalyst with 13.87% CH_4_ conversion *vs.* 3.7% for Ni	111
19	XGBoost regression (with DFT data)	Predict CO_2_RR activity of g-C_3_N_4_ SACs	Identified 11 SACs with *R*^2^ > 0.93; dopants like Zr, Co, Si, Ni showed high selectivity	112
20	Residual U-net (R-U-Net)	Predict CO_2_ plume migration under varying permeability/injection parameters	High-fidelity spatio-temporal plume predictions	113
21	Autoencoder–decoder + multi-dimensional scaling	Visualize evolving CO_2_ plumes from field pressure/temperature data	Generated 3D onset-time plume images	114
22	U-LSTM-net	Integrate spatio-temporal info for multi-field learning and prediction	Outperformed U-Net/Attention U-Net; robust memory and improved dynamic predictions	115
23	MARS (multivariate adaptive regression splines)	Filter-based data assimilation for leakage uncertainty quantification	Optimized measurement locations and reduced leakage uncertainty	116
24	3D recurrent R-U-Net proxy	Predict flow and geomechanical responses; inverse permeability/porosity estimation	Accurate geomechanical responses and parameter estimation from sparse surface data	117
25	Wide ResNet + R-U-Net within ES-MDA	Concurrent estimation of pressure history and plume extent	Produced reliable pressure and saturation maps	118
26	FNO-based proxies + ES-MDA-GEO	History matching to update permeability fields	Accelerated forward modeling with feature coarsening	119
27	R-U-Net within MCMC history matching	Posterior estimation of storage system meta-parameters	Accelerated Bayesian posterior estimation	120
28	PINNs (and HPDNN variants)	Physics-constrained learning with sparse data and PDE inversion	Handled sparse data; extensions to density-driven flow and shale models	121
29	XGBoost proxies	Reservoir response prediction for well placement/control under geological uncertainty	Enabled optimization in WAG operations	122
30	DL proxies + wellbore ROMs	Post-operation leakage assessment using reservoir inputs	Predicted CO_2_/brine leakage factors with TOUGH2-driven reduced models	123
31	AutoKeras-based proxy + classification sub-proxy	Predict wellbore CO_2_/brine leakage rates and classify leakage presence	Provided leakage rates and occurrence classification in coupled reservoir-well models	124

In AI-driven materials modeling, each physicochemical descriptor, such as pore size, surface area, binding energy, or functional group density, serves as an information-bearing feature that encodes the structural and electronic characteristics governing CO_2_ interaction. In information theory, we can measure how strongly a feature *X*_i_ is related to a property *Y* using mutual information. Mutual information tells us how much knowing *X*_i_ helps us predict *Y*. If knowing *X*_i_ reduces a lot of uncertainty about *Y*, then their mutual information is high.^125,126^

### 6.1 Carbon capture and AI/ML

#### 6.1.1 Materials-level models (fingerprints, GNNs, descriptors)

**Fig. 11:**
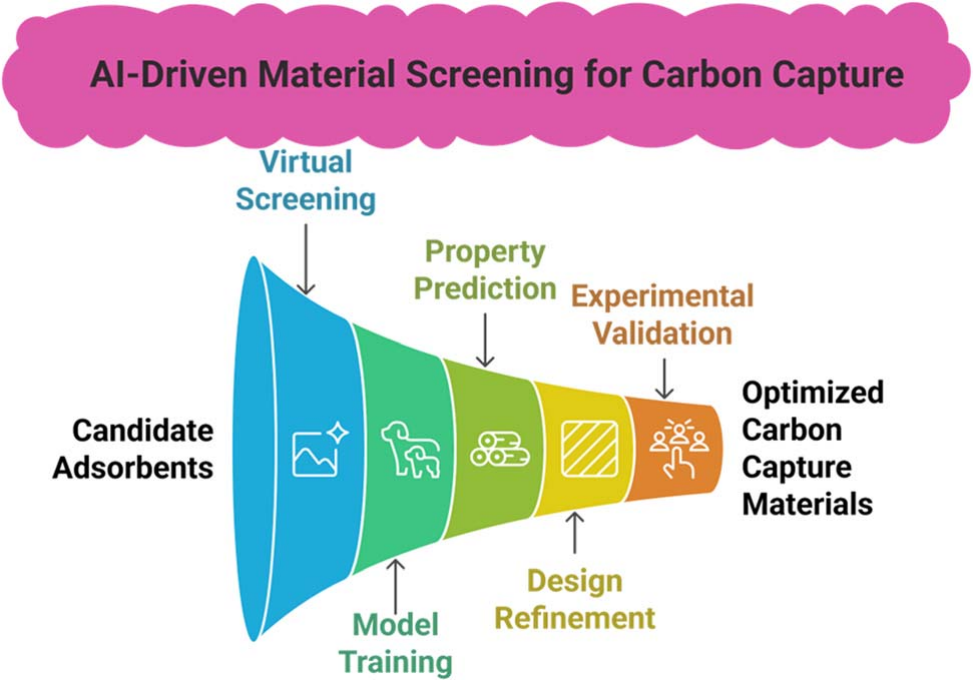
Material screening for carbon capture by using AI/ML.

AI-driven, high-throughput virtual screening (Fig. 11) evaluate thousands of candidate adsorbents, *e.g.*, MOFs and ionic liquids, by predicting CO_2_ adsorption capacity and selectivity before laboratory synthesis using various models like graph neural networks (GNN) and AutoML to automatically optimize structure–performance mappings from large chemical spaces, thereby markedly shortening R&D cycles and lowering costs.

AI also accelerates material design by identifying pore chemistry and topologies through the training of various GNN/AutoML models, validating top candidates, refining design features, and integrating shortlisted materials into models.^97^ For example, an ML-accelerated workflow (GCMC + ML prediction + mixture simulation) screened nearly 268 687 COFs and identified that 3D COFs with approximately 1.0 nm pore size and 2D COFs containing imide linkages and fluoro groups between aromatic rings are the most effective adsorbents for CO_2_.^98^ Thermophysical properties of porous materials imperatively contribute to the adsorption capacity. ML models trained on DFT achieved high accuracy for heat capacity prediction, *e.g.*, 95% for zeolites, with low errors for MFs (3.2 J kg^−1^ K^−1^) and COFs (2.5 J kg^−1^ K^−1^), which enables rapid down-selection and process-informed material design for CCUS.^99^ In another study, self-supervised deep learning generated material embeddings from >120 000 inorganic crystal structures to uncover structure–function relationships, while ML models using property-labeled descriptors predicted thermal properties to accelerate screening for CO_2_ capture materials.^100^ CO_2_ capture performance, along with CO_2_/N_2_ selectivity by MOFs, can also be predicted using an ANN-based ML approach. These models are stable; however, capacity predictions are more accurate than selectivity predictions. Another finding of this model is that surface area and pore size positively correlate with CO_2_ adsorption, but chemical complexities exhibit a slightly negative relationship to CO_2_ adsorption capacity.^101^ AI is considered very crucial in addition to computational chemistry, as MD and DFT can generate synthetic data but face precision and cost limits: ML models trained to refine DFT energy calculations reduce cost while improving accuracy and scalability to larger systems.^102^

#### 6.1.2 Benchmark datasets and validation studies

Back-propagation neural networks (BPNN) and radial basis function neural networks (RBFNN) models were developed using the CO_2_ dataset for different amine solvents (diethanolamine-DEA, methyldiethanolamine-MDEA, *etc.*), achieving good accuracy with experimental values but showing acceptable errors. However, BPNN showed better results than RBFNN. It also performed better than 8 other literature models.^103^ The efficiency of BPNN was further validated by Zhang *et al.* (2018), who developed BPNN and general regression neural networks (GRNN) to demonstrate the potential of AI/ML in predicting adsorbent and solvent properties, as well as CO_2_ solubility within solvents, density, and viscosity. They utilized 433 datasets from previous literature and demonstrated that BPNN is the most effective model for predicting the properties of these solvents. Their findings were further strengthened by showing that GRNN achieved even higher precision for the estimation of the density and viscosity of potassium lysinate and its different mixtures.^104^ To further accelerate the screening of ionic liquids (ILs), over 10 000 experimental datasets from 185 ILs were measured by ML models at varying conditions. By incorporating semi-empirical geometrical and charge-based molecular descriptors, the ensemble random forest (mean absolute error = 0.04) and single decision tree (mean absolute error = 0.10) models achieved a strong correlation with experimental data. This method was more accurate than the quantum-chemistry-based COSMOtherm model for predicting the relation between CO_2_ solubility within ILs.^105^

To address the issue of high costs associated with ILs, ML models (LSSVM, DT, RF, and MLR) were designed using two sets of data from the literature, based on thermodynamic properties and structural descriptors. HOMO–LUMO fractions, along with cationic dispersion, were identified as key variables by GA-MLR. RF and DT also showed valuable results that showed that pressure is one of the influential factors for CO_2_ adsorption.^106^

The above-discussed AI/ML are used to address the concept that how different AI technologies are used in carbon capture methods. Below are some of the AI/ML models that are used in different carbon capture processes and have shown valuable results.

#### 6.1.3 AI/ML in different carbon capture processes (pre-combustion, post-combustion, and direct air capture)

Adsorption, solvent, and gas-separation studies have revealed that ANN, ANFIS, and GA/PSO frameworks can be adapted to syngas conditioning and CO_2_/H_2_ separation for pre-combustion carbon sequestration.^127^ Membranes are a viable option for separating H_2_/CO_2_ in pre-combustion, and AI/ML approaches, including random forest with algorithms for polymer screening, gradient-boosting models for evaluating ∼300 000 MOFs, and neural networks for ion liquid and mixed-matrix membranes, can assist in optimizing material selection and separation performance.^102^

AI allows rapid screening of adsorbents, prediction of CO_2_/N_2_ selectivity, and adsorbent cycle optimization. Likewise, AI assist in the development of more effective and less expensive capture systems.^128^ An example is a techno-economic analysis of PVSA cycles, demonstrating the IISERP MOF-2 developed adsorbent material. It was found that compared to previous studies, a 4-step VSA resulted a capture cost of €33.6 per tonne CO_2_, but during an integrated optimization of the 4-step and a 6-step PVSA cycles across multiple flue-gas CO_2_ compositions, cost savings were 8–35%, while the CO_2_ concentration was ≥7.5% compared to MEA. This example illustrates how coupling advanced adsorbent materials with process design can greatly enhance the economic viability of post-combustion CO_2_ capture.^129^ In another study an AI/ML-enabled workflow was investigated for designing CO_2_ selective polymer membranes for post-combustion capture. Using a polymer property prediction engine trained on experimental data, polymer properties, including CO_2_ permeability, glass transition temperature, and thermal stability, were predicted. Then, an inverse materials design engine proposed new monomer options based on those properties. Out of 784 ML-designed candidates, 390 (∼50%) achieved all targets simultaneously. Meso-scale molecular dynamics simulations are also correlated with membrane performance and showed good quantitative agreement with predicted results. This entire process highlights how AI/ML can expedite the discovery and development of polymer membranes with relevant performance metrics for realistic post-combustion CO_2_ separation conditions.^130^ Recent studies have implemented AI and deep learning to optimize MEA-based post-combustion CO_2_ capture, using CNNs (Convolutional Neural Network), ANNs (Artificial Neural Network), and DBNs (Deep Belief Networks) to generate models that more accurately capture the complex nature of nonlinear processes relative to traditional modeling approaches. Creating Bayesian networks helped to define the main operational parameters, such as lean solvent temperature and flow rate, that can be manipulated to help tune the process. The integrated CNN + Bayesian process has effectively been modeled and optimized the complete system, enabling surrogate modeling and predictions of CO_2_ capture capacity or thermodynamic properties, subsequently allowing improved operational efficiency and decision-making to advance carbon-neutral CCUS strategies.^131^ Another paper applied AI/ML models (DT, RF, XG, SV, and DL modeling) to predict the energy and cooling utilities (SRD, SCD, and SLAD) in amine-based post-combustion CO_2_ capture technologies. Errors in SRD (Sum of Ranking Differences) predictions were in the range of 0.4–3.6% and DL was the most robust method (MAPE < 7%). A SHAP analysis showed that the main factors affecting utility use are the absorber height and the temperature differences (LMTD) in the lean and rich heat exchangers. Moreover, screening of the 6000+ process configurations demonstrated AI/ML can assist in not only optimizing design and reducing operational costs, but also increasing the efficiency of CO_2_ capture.^132^ Additionally, the application of models such as ANN, ANFIS (Adaptive Neuro-Fuzzy Inference Systems), CNN, RBFNN (Radial Basis Function Neural Network), SVM (Support Vector Machine), GA (Genetic Algorithm), PSO (Particle Swarm Optimization), and Bayesian networks affirms the post-combustion CO_2_ capture processes with better predictive capabilities for capture efficiencies, CO_2_ production rates, thermodynamic properties, improved modeling for nonlinear interactions, optimized solvent and adsorption parameters, improved measurement of flow-rate, and pinpointing of the changed operational variables that fully enhance operation strategies for improved efficiencies.^127^

To further illustrate the relevance of the use of AI models, multi-layer perceptrons (MLPs) have been shown to accurately predict solid–vapor and vapor–liquid equilibria in cryogenic direct air capture (DAC) systems. The MLP demonstrates high accuracy compared to traditional thermodynamic models (*R*^2^ ∼ 0.996–0.999), along with reductions in computation time of seven orders of magnitude, enabling real-time CO_2_ control in HVAC integrated systems as part of DAC operations. Overall, these studies illustrated the potential use of AI-enabled phase-equilibrium modeling as a fast and reliable intelligent carbon capture and control strategy for building ecosystems, such as HVAC systems.^133^

These studies, in general, demonstrate that AI and ML represent flexible and accurate methods in pre-combustion, post-combustion, and direct air capture approaches to enhance the pace of material discovery, affirm optimal process design, facilitate real-time controls, and achieve substantial decreases in costs and energy needs, that ultimately improve the performance and affordability of carbon capture technology.

### 6.2 Carbon utilization and AI/ML

#### 6.2.1 Process-level surrogates and digital integration

**Fig. 12:**
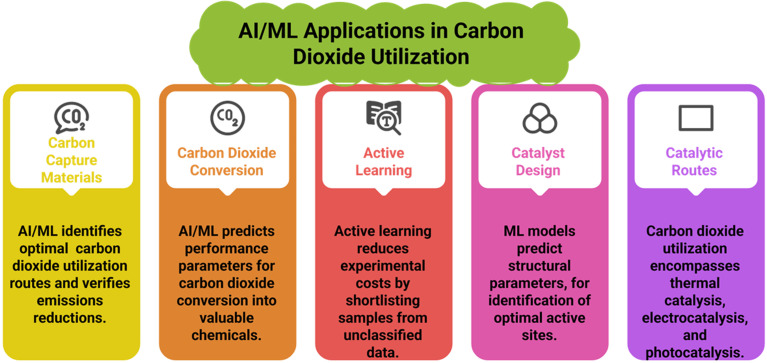
An overview of AI/ML tools for carbon utilization technology.

Apart from CO_2_ capture materials, AI/ML also demonstrates how digitalization, hybrid models, IIoT (Internet of Things), digital twins, and PSE 4.0 can identify optimal CO_2_ utilization routes, integrate them from plant to supply chain, and verify real emissions reductions, thereby de-risking and scaling CCUS deployment.^134^ For example, 2916 cases with nearly 92% accuracy were predicted with a proxy ML model for CCS/EOR optimization. A co-optimized ML system for CO_2_ sequestration and enhanced oil recovery (EOR) stored up to 94% of CO_2_, with 8.74% improved oil production.^101^ For more effective CO_2_-EOR, ANN, SVR, and XGBoost are surrogate models for optimizing several injection strategies and predicting economic outcomes, resulting in more accurate results than traditional AI simulations. Time-of-flight is considered a key aspect for predicting CO_2_ storage, incremental oil production, and revenue metrics. In this regard, random forest is the best feature for cumulative CO_2_ injection.^107^ Several recent CCUS and CO_2_-WAG (water-alternating-gas) studies have used random forest and stacking/ensemble surrogate approaches for injection optimization and performance prediction, showing superior predictive skill for storage volumes and cumulative injection metrics in a field-scale proxy workflow.^135^ Another research introduced a machine-learning workflow that enhanced the optimization of injector well placement for waterflooding, CO_2_ EOR, and storage, while accounting for geological uncertainty. Using an ensemble of geo-models, spatial clustering, and a meta-learner proxy, they can define robust injection scenarios with high predictive accuracy (*R*^2^ > 90%), reducing the time to analytics from hours to minutes.^136^ In another study, a combined clustering-random forest approach was utilized to identify the values for the reactor variables, *i.e.*, catalyst concentration, solvent species, vapor flow rate, and reactor temperature that maximized CO_2_ hydrogenation to C_5+_. The machine learning approach allowed the identification of a high-performing parameter space (∼20–30 bar, 300–340 °C) with 41% CO_2_ conversion with around 40% C_5+_ selectivity, providing insights into the size of crystallite, temperature, and reduction time. This work explained how ML can take data from a heterogeneous literature review and help unveil behavior for selective hydrocarbon production potential for long-chain hydrocarbons.^137^ A machine-learning co-optimization framework coupling ANNs/proxy models with optimization algorithms demonstrated large increases in stored CO_2_ and improved oil recovery (stored ≈94% of injected CO_2_; oil +8.74%).^138^Fig. 12 illustrates some applications of AI/ML in carbon utilization.

#### 6.2.2 AI/ML in catalysis and conversion pathways

For the conversion of CO_2_ into valuable chemicals, AI/ML can play a very pivotal role. For example, solid oxide electrocatalysis cells (SOECs) carry out the electrocatalysis of CO_2_/H_2_O at elevated temperatures for fuel and chemicals generation.^139^ Multi-physics simulations and deep learning algorithms predict SOEC's performance parameters under different thermal parameters.^108^ Chen *et al.* (2020) also employed Extreme Gradient Boosting Regression (XGBR) to predict and screen an electrocatalyst for CO_2_ reduction.^109^ Similarly, DFT with active learning can identify Cu–Al catalysts for CO_2_ conversion to ethylene.^110^ In another study, data-driven high-throughput screening (combining DFT and ML) proposed a Cu–Al catalyst for CO_2_RR (experimentally validated for ethylene production), demonstrating how ML models trained on DFT descriptors can lead to effective experimental outcomes.^140^ In a previous work, XGBoost/ensemble regression models have been used to predict catalytic descriptors (*e.g.*, Δ*G*-CO) and screened large candidate sets with substantial cost savings compared with brute-force DFT. Several studies reported that XGBR can successfully prioritize promising electrocatalysts.^141^ For SOECs, recent multi-physics + ML workflows use physics-based simulations to generate training data and deep learning surrogates to predict performance across temperature/voltage/feed conditions. These combined approaches accelerate design and parameter studies.^142^

#### 6.2.3 Data-collection and active learning loops

Active learning is a framework that provides sampling schemes of training data for machine-learning algorithms, mainly by proactively selecting what data points are necessary to build a precise statistical model in an efficient way.^143^ Active learning considerably brings down experimental/computational costs by shortlisting samples from unclassified data, utilizing many acquisition tools and functions, using new and advanced labels for retraining models, and arresting the process once budget limits are met. On the other hand, strategies like uncertainty sampling method usually target uncertain predictions. Moreover, deep learning models become overconfident when integrated with active learning.^144^ Integrating active learning with DFT, studies show that active learning reduces DFT/experimental budget while identifying high-performance CO_2_RR catalysts. Workflows published on PubMed demonstrate end-to-end AL + DFT discovery with experimental validation.^145^ ML-accelerated DFT screening (with AL) for Cu-based and HEA (High Entropy Alloy) catalysts has been used to target CO_2_ → C_2_ products, confirming the practical gains in screening throughput.^146^ Combining active learning with DFT databases and experiments can reduce the number of calculations or tests needed to find the best candidates. Studies show that active learning workflows can smartly select the most valuable DFT calculations and then confirm the top results experimentally, cutting costs while still finding the best materials.^145^

#### 6.2.4 Reproducibility and explainability in AI models

Despite all the advantages of AI/ML, many ML models are considered as black boxes. Still, explainable AI (XAI) models, as seen in DTs, RF, and XGBoost, where feature importance analysis is carried out using impurity measures, and SHAP (SHapley Additive exPlanations) values provide important chemical insights. However, experimental and scientific domains are still crucial, along with algorithmic aid.^125^ Recent CCUS-related studies have explicitly combined XAI tools (SHAP, feature importance) with ML models to interpret adsorption predictors, catalyst descriptors, and storage prospects, thereby improving interpretability.^147^ In a relatable work, SHAP or similar *post hoc* XAI methods have been applied to adsorption and catalyst ML models to reveal which descriptors (pore size, surface area, electronic features) drive predictions, aiding mechanistic understanding and highlighting potential modes that can lead to failure of system.^148^ Applications of XAI to brine/rock CO_2_ solubility and subsurface storage prediction have been published, demonstrating that explainability increases trust and helps in detecting domain-shift/physics conflicts before field deployment.^149^

#### 6.2.5 Predictive and experimentally validated models

To overcome the limitations of SACs, machine learning (ML) models trained on density functional theory databases can predict structural parameters, such as coordination numbers, ligand types, and bond distances/angles, thereby accelerating the identification of optimal active site geometries. By integrating large DFT datasets with ML algorithms, valuable structure–performance relationships can be established, which are critical for catalyst design.^150^ In the context of CO_2_ utilization, AI has already shown strong potential in accelerating the exploration of catalyst materials.^151^

For instance, copper is an emphatic electrocatalyst for CO_2_ reduction to ethylene, yet the vast number of copper-containing crystals and coordination environments renders full-scale DFT simulations impractical. To address this, Zhong *et al.* (2020) used DFT data as training input for ML regression models and successfully identified a Cu–Al alloy catalyst with a faradaic efficiency exceeding 80%, compared to only 66% for pure Cu catalysts.^110^

Similarly, Sun *et al.* (2024) used DFT-calculated C–H dissociation barriers for surfaces of single-atom alloy (SAA) catalysts as training data for their ML models. After screening over 10 000 candidate structures, they identified an Ir/Ni surface catalyst that demonstrated a 13.87% conversion of methane at 450 °C and 1 atm, compared to 3.7% with pure Ni, solving the deactivation issue related to carbon deposition.^111^ Another study used DFT calculations and an XGBoost regression model to evaluate 11 single-atom catalysts (SACs) supported on g-C_3_N_4_ for CO_2_ reduction. They achieved very high accuracy (*R*^2^ > 0.93) in predicting activity. The study also found that these AI-designed catalysts work well for syngas production, and that certain dopants, like Zr, Co, Si, and Ni, can give high selectivity for CO_2_ reduction.^112^

Utilization of CO_2_ involves numerous catalytic routes such as thermal catalysis, electrocatalysis, and photocatalysis. These can produce various products, like ethylene, methane, and syngas.^152^ However, currently, only the electrocatalytic reduction of CO_2_ and formic acid may have low costs than that of ongoing market prices trends,^153^ which is insufficient to promote CCUS technologies to be adopted on a large scale. So, SACs represent an interesting opportunity for CO_2_ utilization,^154^ particularly when paired with AI predictive modeling to accelerate the catalyst design process.^155^

Data availability is a significant bottleneck, as reliable AI models require thousands of DFT simulations, resulting in substantial computational demands. However, KNR, RFR, SVR, GBR, XGBR, DNN, and GPR are some ML algorithms that can enhance catalyst optimization.^156^

### 6.3 Carbon storage and AI/ML

#### 6.3.1 Process-level surrogates (PINNS, LSTM, FNO)

**Fig. 13:**
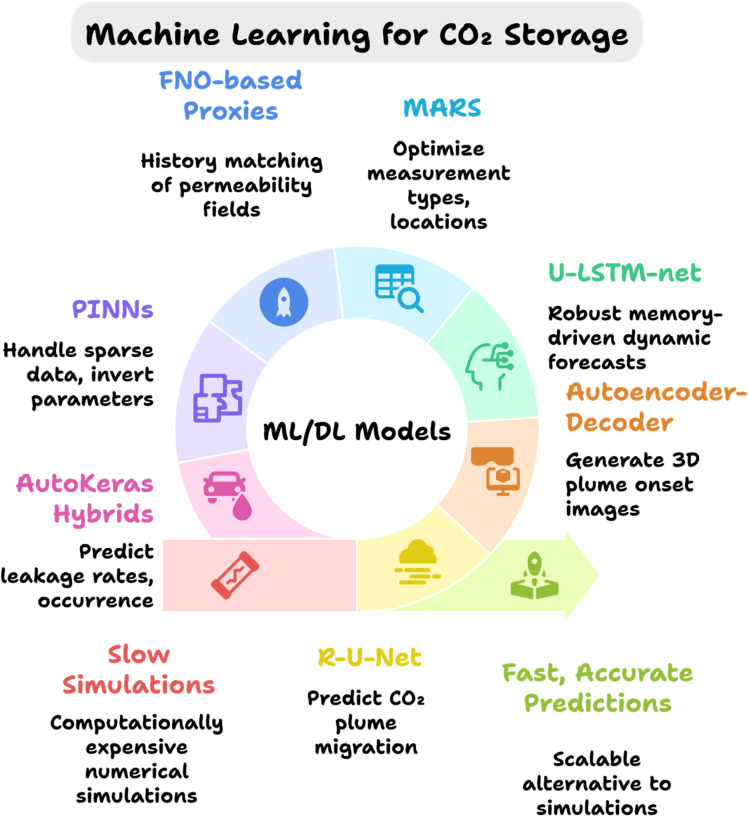
An overview of AI/ML for carbon storage.

Recent advances in machine learning (ML) and deep learning (DL) have greatly accelerated the development of surrogate and proxy models for predicting CO_2_ storage dynamics, plume migration, and leakage risks, providing fast, accurate, and scalable alternatives to computationally expensive numerical simulations. Fig. 13 provides an overview of various AI/ML tools and models used in carbon storage.

The Residual U-Net (R-U-Net) was among the first to demonstrate the capacity to predict CO_2_ plume migration patterns under varying permeability and injection parameters, achieving high-fidelity spatio-temporal outputs.^113^

To extend predictive insights into the visualization domain, an autoencoder–decoder framework coupled with multi-dimensional scaling was introduced, enabling the generation of 3D onset-time images of evolving CO_2_ plumes based on pressure and temperature field data.^114^

To better capture time-based patterns, the U-LSTM-net combined spatial and temporal features for predicting multiple fields. Using transfer learning and GradNorm, it performed better than U-Net and Attention U-Net, providing accurate, memory-aware dynamic forecasts.^115^

Fan *et al.* (2024) explained spatio-temporal forecasts of CO_2_ plume migration using deep networks with transfer learning and interpretation analysis (applies transfer learning/interpretation to U-Net variants).^157^ Wen *et al.* (2021) introduced an R-U-Net-based surrogate for CO_2_ plume prediction, which encodes injection parameters as image channels and predicts spatiotemporal saturation/pressure fields.^113^

#### 6.3.2 Uncertainty and validation in storage modeling

Statistical methods such as Multivariate Adaptive Regression Splines (MARS) contributed to uncertainty quantification by being embedded in filtering-based data assimilation frameworks, where they optimized measurement types and locations to reduce CO_2_ leakage uncertainty.^116^

To accelerate forward modeling, Fourier Neural Operator (FNO)-based proxies, combined with ES-MDA-GEO, were deployed for the history matching of permeability fields, utilizing feature coarsening to enhance computational efficiency.^119^

Physics-informed approaches provided another dimension of robustness. Physics-Informed Neural Networks (PINNs) and their high-performance deep neural network (HPDNN) variants imposed governing PDE constraints directly into the learning process, demonstrating the ability to handle sparse data and invert subsurface parameters.^121^

Chen *et al.* (2018) integrated Multivariate Adaptive Regression Splines (MARS) proxies into filtering/data-assimilation frameworks to optimize monitoring design and reduce CO_2_ leakage uncertainty.^116^ Recent ArXiv and EES reports demonstrate Fourier Neural Operator (FNO)/nested-FNO models for real-time, high-resolution geological CO_2_ storage prediction, and combined workflows (FNO + ES-MDA-GEO) were proposed for fast history matching of permeability fields.^158^ Shokouhi *et al.* (2021) also reported physics-informed deep learning for CO_2_ storage site prediction and inverse problems (PINN frameworks for subsurface flows).^159^

#### 6.3.3 Benchmarking, reproducibility, and future trajectories

Although mostly applied to simple or early-stage geological carbon storage (GCS) cases, recent work has extended to density-driven flow and multi-physics shale models, showing a promising direction for research.^160^ Expanding further, AutoKeras-based hybrid proxies combined regression and classification tasks: the regression sub-proxy predicted CO_2_/brine leakage rates through wells, while the classification sub-proxy identified leakage occurrence across wells and caprock within coupled reservoir-well systems.^124^ Mao *et al.* (2023–2024) describe AutoKeras or AutoML-based hybrid proxies that combine regression and classification tasks to predict wellbore leakage rates and classify leakage occurrence across coupled reservoir-well systems.^161^

Together, these sequential advances show a clear trajectory: from early high-fidelity plume predictions, through spatio-temporal integration, geomechanical coupling, and physics-informed generalization, to ensemble learning and system-level proxies, ML and DL methods are steadily transforming CO_2_ storage modeling into a faster, more accurate, and more comprehensive predictive science.^161^

## 7 Future outlook

**Fig. 14:**
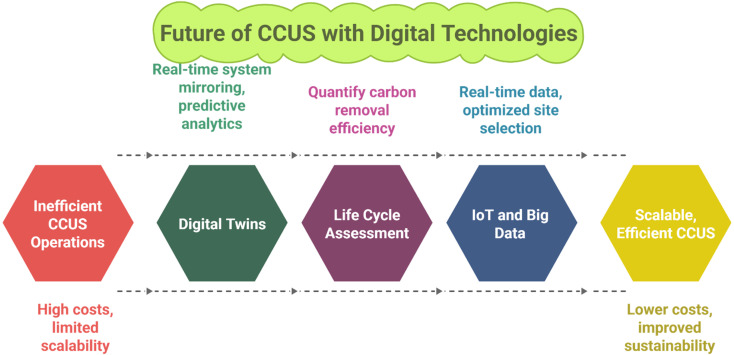
Future outlook of CCUS with digital technologies.

CCUS is a highly promising strategy for achieving a zero-carbon future, and AI/ML has dramatically enhanced its efficiency; however, some areas still require improvement. Digital twins, life cycle assessment (LCA), and IoT are some techniques that can lower the costs of CCUS materials, with expected sustainability as shown in Fig. 14.

### 7.1 Digital twins

Digital technologies are emerging as key components for the future of CCUS, ensuring reliable, scalable, and safe operations. Among these, digital twins hold particular promise for next-generation deployment. As digital, data-based models of CCUS processes, digital twins can mirror real-time system conditions and provide powerful predictive analytics. This capability allows operators to take a proactive approach in terms of performance concerns, mitigate risks, and continuously optimization of various operations through the capture, transport, storage, and utilization processes. They are particularly useful in planning for tomorrow's large-scale CCUS projects as they can simulate “what if” scenarios under complex and uncertain geological conditions. Therefore, if the current commercialization of CCUS is still primarily based on traditional monitoring and control methods, the future of CCUS is more likely to increasingly exploit digital twins as a central tool for predictive maintenance, ongoing tuning, and secure data management. Thus, digital twins can be considered a transformative technology by supporting CCUS toward net-zero carbon goals for tomorrow.^162,163^

### 7.2 Life cycle assessment (LCA)

In the future of CCUS, life cycle assessment will be highly important because it not only characterizes environmental hotspots across CDR (Carbon Dioxide Removal) strategies, such as fuels, polymers, and storage, but also gives clear signals on where systems and policies will need to be developed. Through quantifying carbon removal efficiency, LCA shows that many sites in Europe can likely achieve a CDR efficiency of ≥95%, and offers support to sites and develop energy input in future projects. Most importantly, LCA shows that CCUS often provides a limited contribution to the overall impacts for polymer production and will therefore direct future R&D and investments toward alternative feedstocks and lower carbon energy integration. Collectively, these innovations advance the LCA contribution in becoming a decision-support tool for parliamentarians, policymakers, and industry leaders to help integrate CCUS as an alternative pathway for decarbonization in future decades.^164–166^

### 7.3 IoT and big data analytics

In the near future, the potential of digital technologies (ranging from the Internet of Things (IoT) and big data analytics to automation) for CCUS innovation is noteworthy. Though originally intended to supply chain resilience, these technologies can also be applied to CCUS operations in a way that may enhance environmental and sustainability performance. For example, IoT-based monitoring could facilitate real-time reporting on energy consumption, capture efficiency, and stability of storage. Simultaneously, big data analytics could improve site selection, risk forecasting, and long-term storage monitoring. Automation would help improve the reliability and decrease the cost of complex processes, leading to a more reliable large-scale implementation of CCUS. Furthermore, automated carbon accounting, a technology already used for assessing and managing carbon outputs in supply chains, can be modified to CCUS to provide precise reporting on emissions data, indirect emissions across industrial networks, and transparent and accountable emissions. In sum, convergence of digital technologies in the framework of CCUS represents a forward-looking pathway for scaling up carbon mitigation measures that are efficient and economically feasible.^167,168^

## 8 Conclusion

The pressing issue of addressing CO_2_ emissions requires proficient and scalable solutions, and Carbon Capture, Utilization, and Storage technologies are one of the most viable paths in achieving global decarbonization goals. In this manuscript, we have explored the spectrum of CCUS technologies, including various advanced adsorbent classes like biochar, nanomaterials, graphene derivatives, cellulose nanofibers, and nanoporous carbon, catalysts (including metal–organic frameworks (MOFs), graphene-based electrocatalysis, and single-atom catalysts). While each material offers ways to improve capture efficiency, selectivity, and scalability, they also face challenges in terms of cost, stability, *etc.*, that need careful optimization.

At the same time, innovations in CO_2_ utilization demonstrate that captured carbon can be converted into valuable fuels, chemicals, and materials in a circular carbon economy. New strategies with mineral carbonation, hydrate-based storage, and polymeric membranes accelerate the possibilities of safe CO_2_ sequestration. Importantly, the incorporation of artificial intelligence and machine learning has transformed the research and deployment of CCUS *via* accelerated material discovery, predictive modeling of catalytic pathways, storage dynamics optimization, and system-level decision-making. Enhancements in digital technologies, such as digital twins, IoT monitoring, and life cycle assessment frameworks, represent the beginning of a complete transition towards safer, cheaper, and more sustainable CCUS solutions.

Despite these advances, there are still certain challenges. Scaling laboratory performance to industrial conditions, time-saving, leakage-free storage in terms of environmental integrity and safety, associated unintended consequences, and pathways for commercializing CCUS approaches are some of the main challenges. Surpassing these barriers requires an integration of material innovation and digital intelligence. Hence, CCUS is not a single domain; rather, it is a collection of approaches that require advancements at various levels. Once CCUS technology is developed, it can mitigate many environmental problems. For this to occur, future research must continue to focus on advanced laboratory performance to real-world applications.

## Author contributions

Somia Mazhar contributed to conceptualization, literature investigation, methodology design, drafting of the original manuscript, and preparation of figures. Muhammad Waseem Mumtaz provided overall supervision, conceptual guidance, critical review, and editing, and served as a corresponding author. Mohamed El Oirdi contributed to supervision, content validation, and critical revision, and served as the corresponding author. Hamid Mukhtar assisted with resources, data curation, and manuscript review. Muhammad Asam Raza supported formal analysis and literature organization. Mohd Farhan contributed to literature validation and editing. Mohammad Aatif assisted with data collection, resource compilation, and manuscript review. Ghazala Muteeb contributed through funding acquisition, resources, manuscript visualization, and critical review. All authors read and approved the final version of the manuscript.

## Conflicts of interest

The authors declare no competing interests.

## Data Availability

No primary research results, software or code have been included and no new data were generated or analysed as part of this review.
